# Lightweight UAV-based object detection and tracking for intelligent oil and gas field safety monitoring

**DOI:** 10.1371/journal.pone.0358757

**Published:** 2026-09-25

**Authors:** Jianhua Gong, Yifu Wang, Jun Zhang, Defei Du, Yang Huang

**Affiliations:** Digital Intelligent Technology Subsidiary of Southwest Oil and Gas Field, Chengdu, China; Hiroshima University: Hiroshima Daigaku, JAPAN

## Abstract

Continuous, reliable monitoring of geographically dispersed oil and gas production facilities is essential for accident prevention, asset integrity, and regulatory compliance, yet ground-based inspection is labour-intensive and temporally sparse. Unmanned aerial vehicles (UAVs) offer on-demand aerial coverage, but automated interpretation of production-site imagery is hindered by extreme target-scale variation, irregular equipment geometries, and the limited compute available on airborne platforms. This paper presents an integrated lightweight detection-and-tracking framework tailored to oil and gas field surveillance. The detector augments YOLOv8s with deformable C2f (DCNv2) modules for geometry-adaptive feature extraction, a progressive feature pyramid (AFPN) that suppresses cross-scale semantic conflict, a parameter-shared detail-enhanced head (LSDECD-Head), a Focaler-GIoU regression loss, and LAMP structured pruning for embedded deployment. For dynamic situational awareness, an improved ByteTrack introduces a spatial–appearance similarity matrix encoding operational-state cues and an acceleration-aware Kalman correction. On VisDrone2019, UAVDT, and a self-constructed oil-and-gas dataset (OGF-UAV), the method attains 44.1%, 57.6%, and 56.3% mAP@0.5 with only 7.8 M parameters at 78 FPS, and reduces tracking identity switches by up to 43.2%. The pruned model runs at 18.3 FPS on an NVIDIA Jetson Orin NX 16 GB under TensorRT FP16, confirming practicality for real-time field deployment.

## 1. Introduction

Oil and gas production sites are spatially dispersed, operationally hazardous environments in which leaking valve assemblies, overheating pipeline segments, and unauthorized vehicle or personnel incursions can rapidly escalate into safety incidents, production losses, and environmental damage. Throughout the production cycle, continuous surveillance of this widely distributed surface infrastructure underpins accident prevention, asset integrity, and regulatory compliance, yet conventional ground-based inspection is labour-intensive, temporally sparse, and unable to deliver the timely visual evidence that proactive monitoring demands. Rotary-wing unmanned aerial vehicles (UAVs) equipped with high-resolution visible-spectrum and thermal cameras address these limitations by acquiring multi-perspective imagery on demand, capturing fine-grained equipment detail and wide-area situational context within a single flight over terrain that is difficult or dangerous to survey from the ground. Coupled with advances in machine-learning-based image interpretation, such platforms establish a compelling aerial inspection paradigm for petroleum operations—capable of on-demand deployment, simultaneous multi-perspective sensing, and adaptive analysis of complex production scenes [1,2].

Yet translating the promise of aerial monitoring into reliable automated perception remains non-trivial. Three interrelated difficulties stand out. First, the diversity of target scales in oil and gas field imagery is extreme, spanning sub-10-pixel worker silhouettes to multi-hundred-pixel storage tanks within the same frame. Conventional feature pyramid architectures, while effective for moderate scale variation, tend to lose critical fine-grained detail when fusing non-adjacent level features directly [3]. Second, the irregular geometries of petroleum infrastructure—curved pipelines, corrugated tank surfaces, articulated drilling rigs—challenge standard grid-sampled convolution kernels, which implicitly assume that nearby pixels share similar semantics [4]. Third, onboard compute budgets on medium-class commercial drones (payload ≤ 500 g) are typically limited to single-board processors such as the NVIDIA Jetson Orin or equivalent, where state-of-the-art detectors with tens of millions of parameters would saturate memory bandwidth and preclude real-time operation [5].

Alongside static detection, continuous tracking of moving entities—maintenance vehicles, personnel, supply trucks—is essential for situational awareness and anomaly detection in dynamic oil field scenarios. Multi-object tracking (MOT) algorithms must sustain consistent identity assignment across frames even when targets are temporarily occluded by structural elements or other vehicles, and when appearance cues are degraded by shadows, dust, or heat shimmer near flare stacks [6]. Standard tracking-by-detection pipelines such as ByteTrack [7] and OC-SORT [8] exhibit elevated ID-switch rates under these conditions, largely because their association metrics do not adequately model acceleration-driven trajectory deviations or exploit functional state information (e.g., loaded vs. unloaded vehicle) as a discriminative cue.

Motivated by these shortcomings, this paper proposes an integrated lightweight detection and tracking system optimized specifically for UAV-based oil and gas field inspection. The detection backbone is derived from YOLOv8s [9] and augmented with a set of targeted architectural modifications. DCNv2 operators [10] replace standard convolutions within the C2f Bottleneck to allow dynamic sampling point adaptation. AFPN [11] replaces the conventional PANet neck to eliminate semantic collisions between non-adjacent feature levels. A purpose-built LSDECD-Head reduces head-level parameter redundancy while maintaining sensitivity to both small and large targets. Focaler-GIoU loss [12,13] applies adaptive sample weighting to concentrate gradient energy on regression-difficult instances. LAMP pruning [14] then compresses the trained model to a size suitable for embedded inference. On the tracking side, a modified ByteTrack extends the original cost-matrix formulation with an ASM that incorporates spatial proximity, visual appearance, and operational state, coupled with an acceleration-corrected state estimator.

The primary contributions of this work are summarized as follows:

(1) We propose a systematically improved YOLOv8s detector incorporating three architectural enhancements: a DCNv2-augmented C2f module for geometry-aware feature extraction, an AFPN neck for progressive cross-scale fusion that eliminates semantic conflicts between non-adjacent feature levels, and a lightweight LSDECD-Head that shares detail-enhancement convolutions across all prediction scales, collectively achieving higher accuracy with reduced parameter count and computational cost.
(2) We derive Focaler-GIoU, a novel loss function that unifies the geometric correction of GIoU with interval-based sample reweighting of Focaler-IoU, redistributing the overlap-driven regression gradient toward the band of samples for which localisation is feasible but incomplete, while retaining a non-vanishing geometric signal on zero-overlap instances.
(3) We augment ByteTrack with a spatial–appearance similarity matrix (ASM) encoding spatial, visual, and operational-state cues, alongside an acceleration-aware Kalman correction term, reducing identity switches by over 43% on VisDrone2019-MOT relative to the standard pipeline.
(4) We introduce OGF-UAV, a self-constructed UAV dataset comprising 2,847 annotated images across six oil and gas field categories, and demonstrate that the proposed framework achieves consistent and significant performance gains on this application-specific benchmark alongside the public VisDrone2019 and UAVDT datasets.

The remainder of this paper is organized as follows. Section 2 surveys related work. Section 3 details the proposed methodology. Section 4 presents experimental results and ablation studies. Section 5 concludes the paper and discusses future directions.

## 2. Related work

### 2.1 UAV-based object detection

Detection from aerial platforms presents unique perceptual difficulties compared with standard ground-level imagery. Early contributions such as TPH-YOLOv5 [15] embedded Transformer prediction heads into a YOLO backbone to capture long-range contextual dependencies, reporting notable gains on the VisDrone2019 benchmark. CEDAM [16] addressed the scale-imbalance problem through a context-enriched feature aggregation module designed for dense crowd scenarios in UAV footage. ClusDet [17] proposed to first cluster the image into region-of-interest sub-windows before applying a secondary detector, trading latency for improved recall on tiny objects. More recent work has explored neural architecture search (NAS) for automatic discovery of mobile-efficient topologies tailored to aerial perception [18,19]. Parallel efforts in other aerial-inspection domains corroborate the primacy of scale handling: Zheng et al. [20] coupled an anchor-free YOLOv11 variant with multiscale feature enhancement for grape yield estimation in precision viticulture, reporting that scale-adaptive prediction, rather than backbone capacity, dominates accuracy on small and densely clustered targets. Although the platform, target semantics and compute envelope of that setting differ substantially from industrial oil and gas inspection, the observation aligns with the design priorities adopted here. Machine- and deep-learning pipelines have likewise been applied to aerial and satellite imagery for land-cover and vegetation mapping tasks, including nanosatellite scene classification [21], UAV-based weed detection [22] and supervised classification of forest areas from UAV imagery [23]. These works address per-scene or per-pixel classification for agricultural and forestry mapping rather than real-time detection and tracking under an embedded compute budget, but they illustrate the breadth of aerial-imagery interpretation problems for which learned representations have displaced hand-crafted features. Despite these advances, the joint optimization of accuracy, compactness, and real-time throughput on embedded UAV hardware remains an open research challenge, particularly under the challenging conditions of industrial oil and gas fields.

### 2.2 Deformable convolution and attention mechanisms

Deformable Convolutional Networks (DCN) [24] introduced learnable per-location sampling offsets into the standard grid convolution, substantially improving sensitivity to geometric transformations. The follow-up DCNv2 [10] further incorporated per-point modulation scalars to down-weight irrelevant background activations, demonstrating improvements on COCO object detection. Subsequent work has embedded DCN within YOLO-family detectors to improve localization of deformable industrial objects [25]. Attention mechanisms, including Squeeze-and-Excitation (SE) [26], CBAM [27], and the more recent ECA-Net [28], have also been widely adopted to recalibrate channel or spatial feature responses. Our approach opts for DCNv2 as the backbone augmentation because it provides implicit spatial attention with negligible hyper-parameter tuning, making it practical for deployment in field applications.

### 2.3 Multi-scale feature fusion

Feature Pyramid Networks (FPN) [29] established the dominant paradigm for hierarchical feature reuse in object detection. PANet [30] augmented FPN with a bottom-up shortcut path to shorten information propagation and demonstrated improved small-object localization. BiFPN [31] introduced learnable weights for each fusion edge, while FPE‑YOLO [32] automated the search for near-optimal connectivity patterns. AFPN [11] revisited the fusion order: rather than aggregating features top-down or bottom-up in a single pass, it progressively merges adjacent levels before introducing higher-order semantic information, thereby avoiding semantic conflicts that arise when non-adjacent feature maps are fused directly. Our adaptation of AFPN into the YOLOv8s neck demonstrates that its progressive fusion strategy is particularly beneficial in aerial inspection datasets where scale diversity spans multiple orders of magnitude.

### 2.4 Lightweight detection heads and loss functions

The detection head accounts for a non-trivial fraction of inference latency in single-stage detectors. GFL [33] and TOOD [34] proposed task-aligned heads that jointly optimize classification and localization, but at the cost of increased module complexity. DAMO-YOLO [35] demonstrated that a carefully designed lightweight head can match or exceed the accuracy of heavier counterparts when combined with an appropriate neck. On the loss function side, IoU-family metrics—GIoU [36], DIoU, CIoU [37]—progressively incorporated geometric penalty terms to address incomplete overlap and aspect-ratio misalignment. Focal loss [38] addressed foreground–background class imbalance by down-weighting confidently classified examples. Focaler-IoU [12] extended this philosophy to the regression domain by selectively suppressing gradient contributions from trivially regressed samples. Our Focaler-GIoU loss integrates the geometric correction of GIoU with Focaler-IoU’s sample weighting, providing a balanced training signal that is particularly effective for the high-difficulty small-target instances common in UAV imagery.

### 2.5 Multi-object tracking

Contemporary MOT research largely follows the tracking-by-detection paradigm [39]. The foundational SORT algorithm [40] coupled a Kalman filter with the Hungarian algorithm for real-time tracking. DeepSORT [41] enriched the cost matrix with a deep appearance descriptor to reduce ID switches under short-term occlusion. ByteTrack [7] improved recall by retaining low-confidence detections in a secondary association stage, achieving a favorable accuracy–speed trade-off. OC-SORT [8] replaced the standard Kalman update with an observation-centric model to suppress error accumulation during occlusion intervals. BoT-SORT [42] further fused camera-motion compensation with appearance re-identification. Orthogonally to this association-centric line of work, spatio-temporal convolutional architectures couple detection with short-horizon motion modelling: Meng et al. [43] report that jointly encoding spatial and temporal cues improves target localization for unmanned pineapple picking. The application domain and platform kinematics differ considerably from UAV inspection, and we deliberately keep detection and association decoupled to preserve the real-time budget, but the underlying observation — that temporal context is a cheap and complementary source of discriminative signal — motivates the acceleration-aware correction introduced in Section 3.7.2. Despite these advances, trackers operating in high-density industrial environments—where many vehicles exhibit nearly identical appearances and frequent mutual occlusions—continue to suffer elevated ID-switch rates. Our modified ByteTrack addresses this gap by augmenting the cost matrix with functional operational-state information and introducing an acceleration-aware correction term into the Kalman state update equation.

## 3. Proposed method

### 3.1 Overall architecture

**Fig 1:**
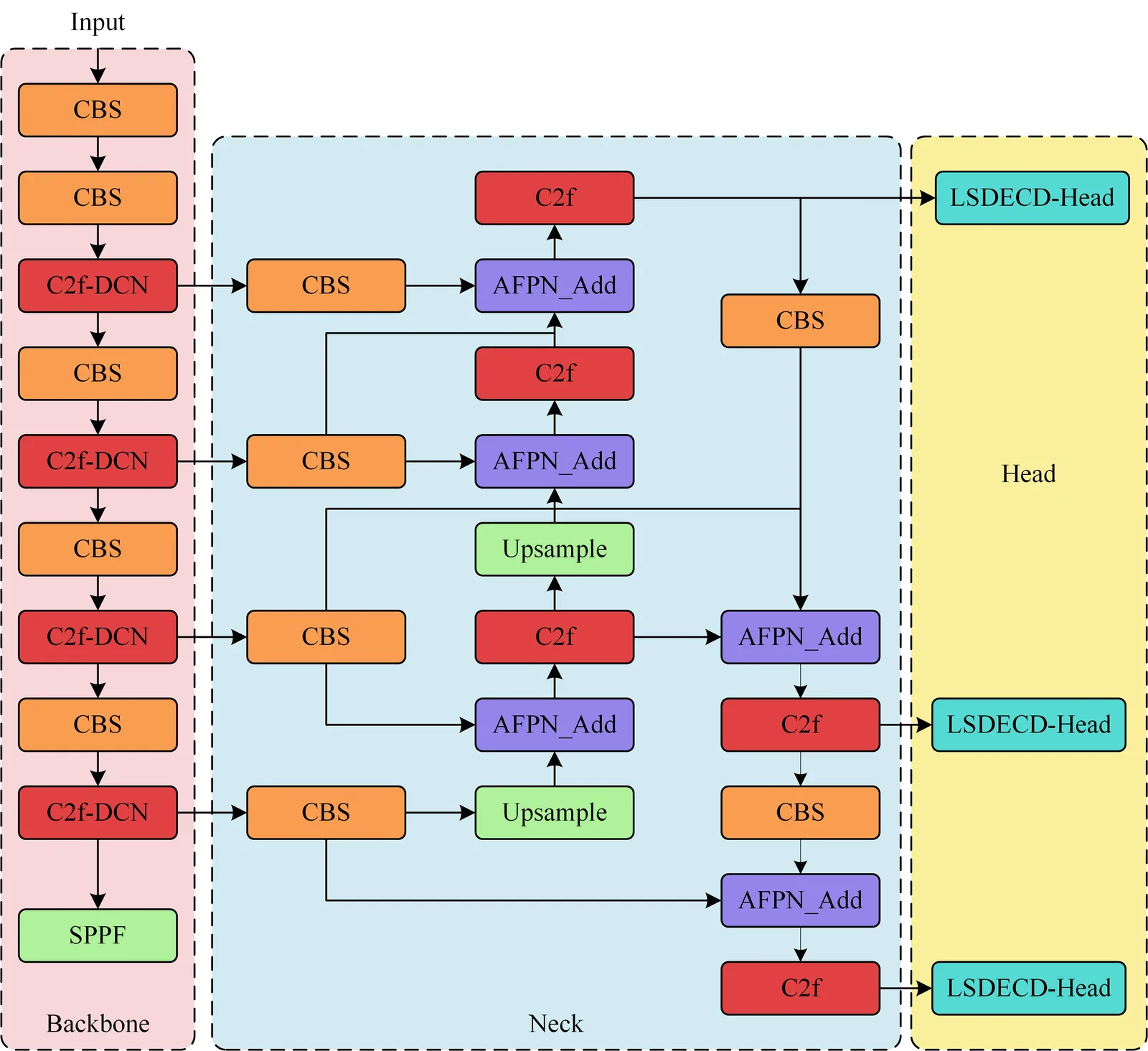
Overall architecture of the proposed improved YOLOv8s detector. The backbone employs C2f-DCN modules for deformable feature extraction; the neck integrates AFPN for progressive multi-scale fusion; and the head uses LSDECD-Head for efficient multi-scale prediction.

The proposed system consists of two principal components: a lightweight object detector derived from YOLOv8s and an improved multi-object tracker. The complete modified YOLOv8s architecture is depicted in Fig 1. The backbone replaces standard C2f modules with DCNv2-augmented variants (C2f-DCN) to enable geometrically adaptive feature extraction. The neck adopts AFPN with progressive feature aggregation nodes (AFPN_Add), superseding the conventional PANet. The detection head is replaced by LSDECD-Head, which processes four multi-resolution feature maps (P2–P5) through shared detail-enhancement convolutions before branching into classification and regression sub-heads. At inference time, the pruned model is loaded onto an NVIDIA Jetson Orin embedded platform for on-UAV processing.

### 3.2 C2f backbone reconstruction with DCNv2

#### 3.2.1 Motivation and deformable convolution formulation.

**Fig 2:**
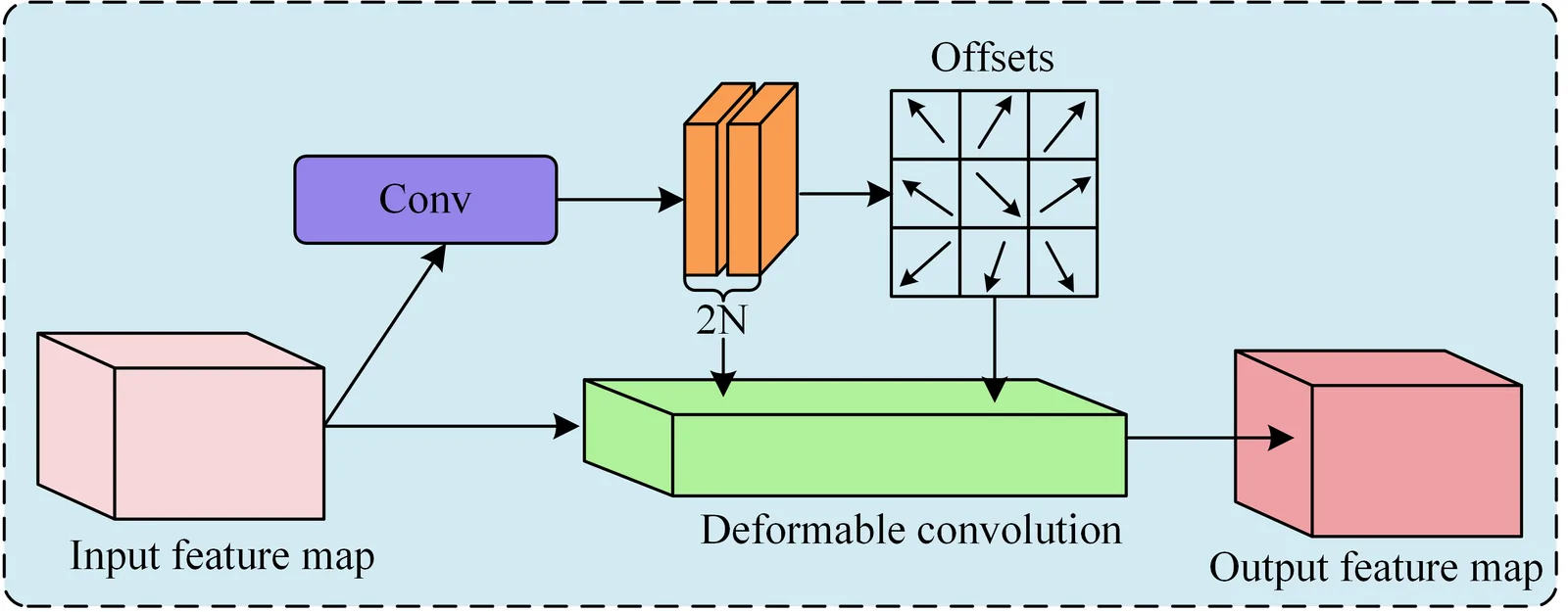
Schematic of the deformable convolutional structure. A parallel convolution branch learns 2N spatial offset values that dynamically relocate the sampling positions of the N point kernel grid, enabling geometry-adaptive feature aggregation.

Standard convolution operations enforce a fixed rectangular sampling grid, an implicit assumption that target shapes are spatially regular. In oil and gas field imagery, equipment such as drilling rigs, separator vessels, and articulated service vehicles frequently exhibits pronounced geometric deformation when observed from oblique UAV viewpoints. To address this limitation, we incorporate DCNv2 into the Bottleneck structure within the C2f module. The fundamental principle of deformable convolution is illustrated in Fig 2.

For a standard 3×3 convolution, the output feature at position $p_{\text{0}}$ is:


$$\begin{array}{c} y\left(p_{\text{0}}\right)=\sum_{p_{n}\in R}w\left(p_{n}\right)\cdot x\left(p_{\text{0}}+p_{n}\right) \end{array}$$ (1)

where R denotes the 3×3 sampling grid, $p_{n}$ is the n -th grid offset, and $w\left(p_{n}\right)$ is the corresponding kernel weight. DCN augments each sampling location with a learned displacement $\Delta p_{n}$:


$$\begin{array}{c} y\left(p_{\text{0}}\right)=\sum_{p_{n}\in R}w\left(p_{n}\right)\cdot x\left(p_{\text{0}}+p_{n}+\Delta p_{n}\right) \end{array}$$ (2)

DCNv2 additionally introduces a per-location modulation scalar $\Delta m_{n}\in \left[0,1\right]$, which serves as an implicit attention gate to suppress activations from contextually irrelevant regions:


$$\begin{array}{c} y\left(p_{\text{0}}\right)=\sum_{p_{n}\in R}w\left(p_{n}\right)\cdot x\left(p_{\text{0}}+p_{n}+\Delta p_{n}\right)\cdot \Delta m_{n} \end{array}$$ (3)

The combined effect of spatial offsets and modulation enables the convolution to focus on discriminative structural features regardless of the underlying morphological variability, which is particularly advantageous for deformable petrochemical apparatus.

#### 3.2.2 C2f-DCN Module.

**Fig 3:**
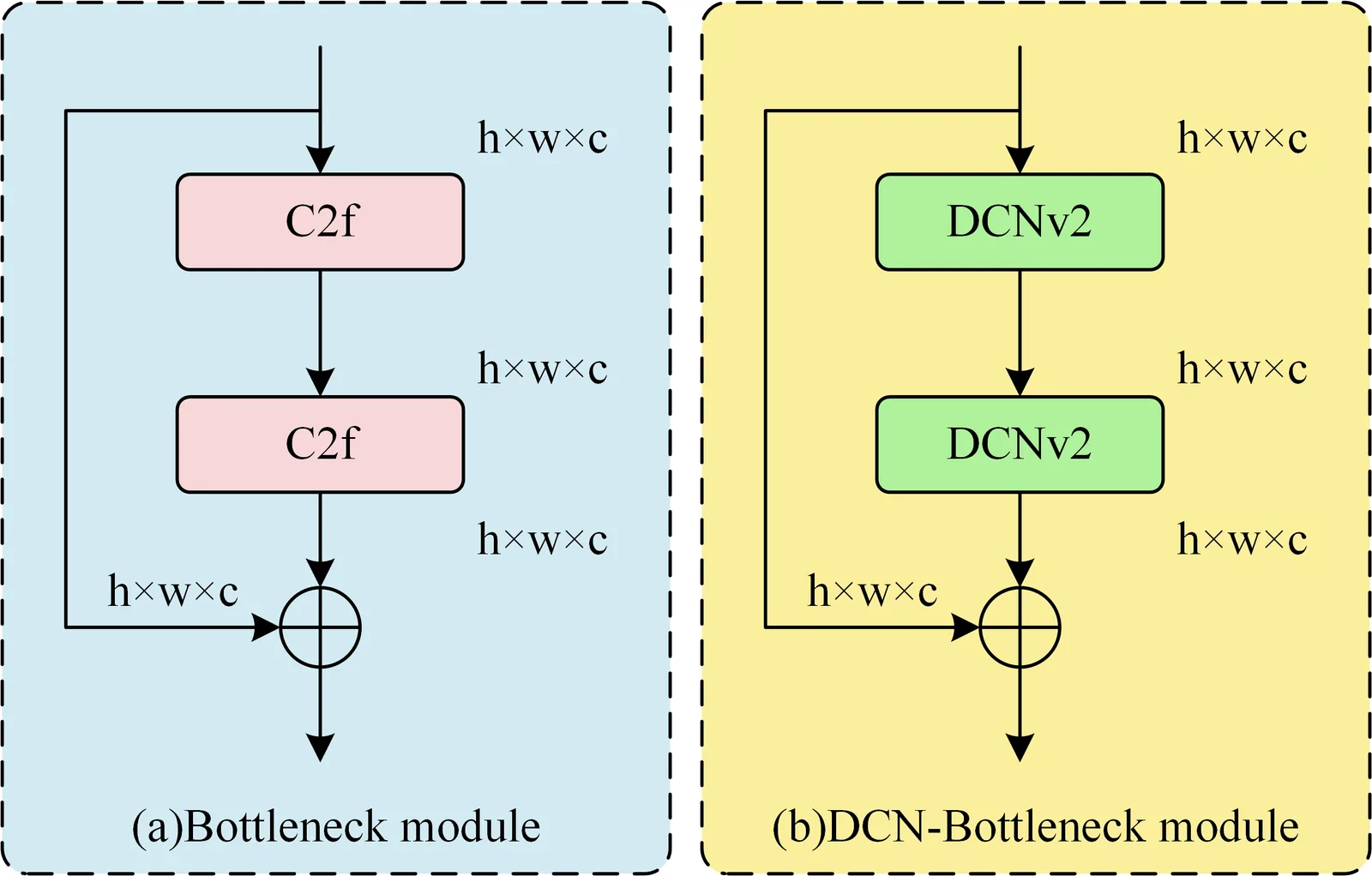
Comparison of the standard Bottleneck module (a) and the proposed DCN-Bottleneck module (b). Standard 3 × 3 convolutions are replaced by DCNv2 operators, enabling adaptive spatial sampling while maintaining the residual identity path.

As illustrated in Fig 3, we replace the two convolutional layers within each Bottleneck block in the C2f module with DCNv2 operators, forming the DCN-Bottleneck. The residual identity shortcut is retained to preserve gradient flow and mitigate the potential optimization instability introduced by the learnable offsets. The offset and modulation branches are implemented as lightweight parallel convolutions sharing the same receptive field as the main branch, introducing approximately 8% additional parameters per module. To prevent excessive offset magnitudes from destabilizing early training, offset values are initialized to zero and subject to gradient clipping at 1.0 during the first ten epochs.

### 3.3 Progressive feature pyramid network (AFPN)

**Fig 4:**
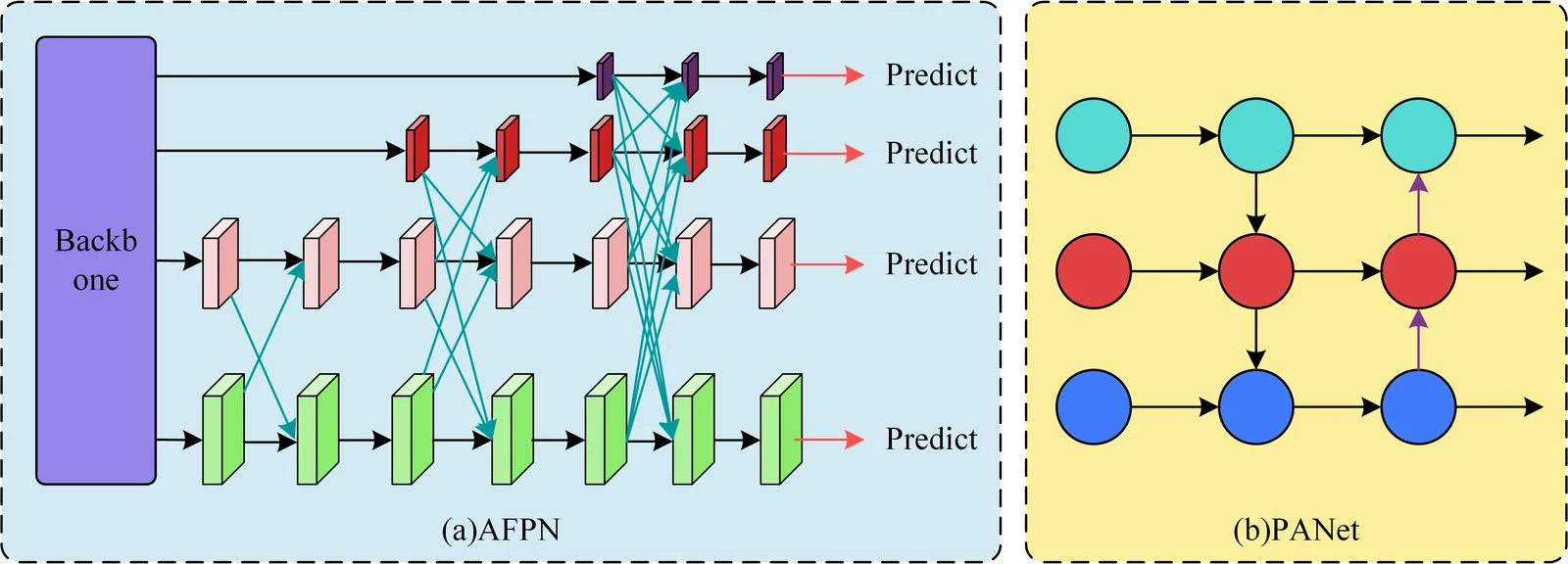
Architectural comparison between PANet (b) and AFPN (a). AFPN progressively merges adjacent feature levels before introducing higher-order semantics, avoiding the semantic conflict caused by direct non-adjacent level fusion in PANet.

The default YOLOv8s neck employs a PANet structure that connects feature levels through top-down and bottom-up pathways. While PANet effectively enriches shallow features with high-level semantic context, direct fusion between non-adjacent levels (e.g., C3 and P5 in a five-level hierarchy) introduces semantic conflicts: low-level texture responses interfere with high-level categorical representations, degrading detection precision in scenes where targets of vastly different scales coexist. Fig 4 contrasts the PANet connectivity with the AFPN structure adopted in our design.

AFPN fuses features only after they have been brought into geometric and channel correspondence. Let $C_{l}$ and $\left(H_{l},W_{l}\right)$ denote the channel width and spatial resolution of the target level l ($C_{\text{2}}-C_{\text{5}}=64,128,256,512$ at strides 4, 8, 16, 32). We define three alignment operators: $\varphi_{\text{k}\to \text{l}}\left(\cdot \right)$, a 1 × 1 convolution with BN and SiLU mapping $C_{k}$ channels to $C_{l}$; ${\text{U}}_{\text{k}\to \text{l}}\left(\cdot \right)=\varphi_{\text{k}\to \text{l}}$ followed by bilinear upsampling with factor ${\text{2}}^{k-l}$; and ${\text{D}}_{\text{k}\to \text{l}}\left(\cdot \right)=\varphi_{\text{k}\to \text{l}}$ followed by (l−k) successive stride-2 3 × 3 convolutions. Every “+” in Eqs. (4)–(7) is consequently an element-wise addition of tensors of identical shape $H_{l}\times W_{l}\times C_{l}$:


$$\begin{array}{c} F_{12}=R\left({\text{D}}_{1\to 2}\left(F_{\text{1}}\right)+\varphi_{2\to 2}\left(F_{\text{2}}\right)\right) \end{array}$$ (4)

$$\begin{array}{c} F_{\text{final}}=R\left({\text{D}}_{2\to 3}\left(F_{12}\right)+\varphi_{3\to 3}\left(F_{\text{3}}\right)\right) \end{array}$$ (5)

Each fusion step passes through a residual refinement unit R(x):


$$\begin{array}{c} R\left(x\right)=x+f_{3\times 3}^{\text{2}}\left(f_{3\times 3}^{\text{1}}\left(x\right)\right) \end{array}$$ (6)

where $f_{3\times 3}^{\text{1}}$ and $f_{3\times 3}^{\text{2}}$ denote consecutive 3×3 convolutional layers. To resolve remaining information conflicts at each spatial position (i,j), AFPN employs an adaptive spatial fusion (ASF) operation. Given three level-specific feature vectors $x_{ij}^{1\to 1},x_{ij}^{2\to 1}$, and $x_{ij}^{3\to 1}$, the fused output is:


$$\begin{array}{c} y_{ij}^{l}=\alpha_{ij}^{l}\cdot x_{ij}^{1\to 1}+\beta_{ij}^{l}\cdot x_{ij}^{2\to 1}+\gamma_{ij}^{l}\cdot x_{ij}^{3\to 1} \end{array}$$ (7)

where the parameters are subject to the constraint $\alpha_{ij}^{l}+\beta_{ij}^{l}+\gamma_{ij}^{l}=1$ for all (i,j). The three ASF inputs are the aligned versions of the feature maps that meet at level l, i.e., $x^{\text{k}\to \text{l}}={\text{D}}_{\text{k}\to \text{l}}\left(F_{k}\right)$ for k<l, $\varphi_{\text{k}\to \text{l}}\left(F_{k}\right)$ for k=l, and ${\text{U}}_{\text{k}\to \text{l}}\left(F_{k}\right)$ for k>l; all three therefore have shape $H_{l}\times W_{l}\times C_{l}$. The fusion weights are obtained by concatenating the three aligned tensors along the channel axis, applying a 1 × 1 convolution with three output channels, and taking a softmax across those three channels, which enforces $\alpha_{\text{ij}}^{l}+\beta_{\text{ij}}^{l}+\gamma_{\text{ij}}^{l}=1$ at every spatial position (i,j) by construction rather than by post-hoc normalisation. Compared with PANet, AFPN reduces neck-level FLOPs by approximately 12\% in our configuration while improving smallobject recall by 2.3 percentage points in ablation experiments, which is attributed to the elimination of conflicting gradient paths during training.

### 3.4 Lightweight shared detail-enhanced detection head (LSDECD-Head)

**Fig 5:**
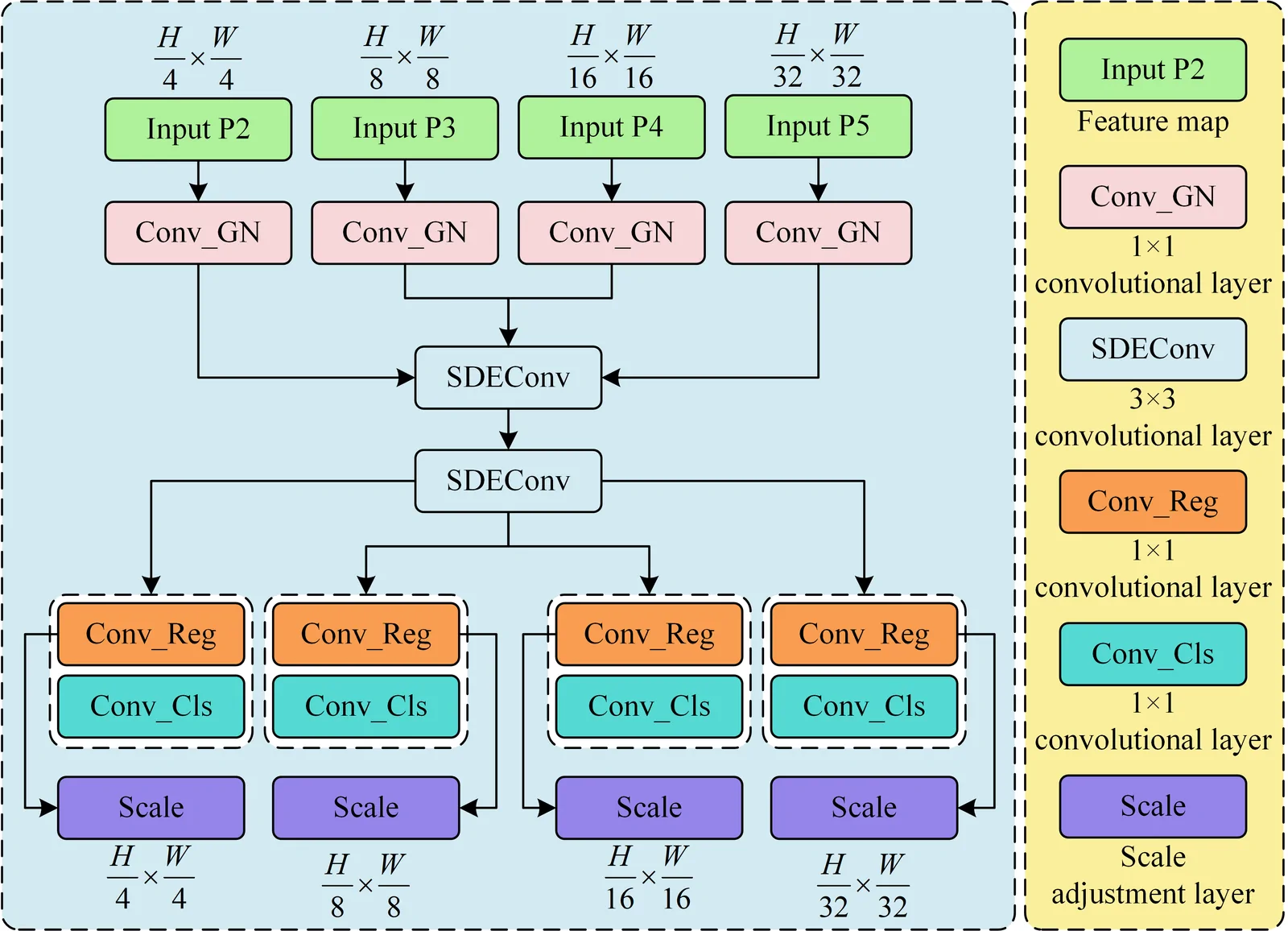
Architecture of the LSDECD-Head. Four multi-scale feature maps are first channel-normalized via 1 × 1 Conv_GN layers, then jointly processed by two shared SDEConv (3 × 3) modules. The resulting features are routed to scale-specific Conv_Cls and Conv_Reg branches, with a Scale layer in the regression branch to compensate for inter-scale bounding box magnitude differences.

Standard YOLO detection heads assign independent convolutional branches to each feature level, resulting in significant parameter duplication across scales. The proposed LSDECD-Head, illustrated in Fig 5, addresses this inefficiency through a cross-scale shared detail-enhancement convolution module (SDEConv) that processes all four input feature maps (P2–P5) jointly before branching into per-scale classification and regression outputs.

Specifically, four input feature maps at resolutions H/4, H/8, H/16, and H/32 are first normalized through independent 1 × 1 group-normalized convolutions (Conv_GN) to align channel dimensions. The normalized maps are then fed into two sequential SDEConv modules, each implementing a 3 × 3 depthwise separable convolution followed by a channel attention gate, shared across all four scales. The shared design reduces head-level parameters by approximately 38% (3.95 M → 2.45 M) relative to a standard YOLOv8 decoupled head instantiated on the same four AFPN outputs. This saving is purely architectural and is measured before any pruning; it is therefore disjoint from, and additive to, the 23.5% reduction later obtained by LAMP (Section 3.6). Per-scale classification (Conv_Cls) and regression (Conv_Reg) branches employ 1 × 1 convolutions, and a learnable Scale layer in the regression branch compensates for the systematic magnitude differences between predictions at different feature pyramid levels. The per-level Conv_GN layers are what make cross-scale sharing viable: they are independent rather than shared, so level-specific statistics are absorbed before the shared SDEConv modules are reached, and group normalisation is used in preference to batch normalisation because the four levels have markedly different activation statistics that per-level running estimates would not transfer across a shared operator. Measured on the VisDrone2019 validation set, the mean cosine similarity between P2 and P5 features rises from 0.31 at the raw AFPN outputs to 0.68 after Conv_GN, and per-level activation standard deviations converge from a range of 0.42–2.71 to 1.00 ± 0.06. Replacing the four independent Conv_GN layers with a single shared one reduces mAP@0.5 from 44.6% to 43.7%, confirming that this normalisation, rather than the shared convolution itself, is what prevents cross-scale misalignment. The scale-aware attention and spatial deformable sampling in SDEConv enable LSDECD-Head to detect targets ranging from pedestrians (≈ 6 pixels tall) to large storage tanks (≈ 800 pixels wide) within a single unified prediction framework. Including the P2 feature map (H/4 resolution) introduces an explicit computational cost: the P2 branch contributes approximately 4.1 GFLOPs of the total 25.4 GFLOPs in the unpruned model (16.1%), primarily from the SDEConv depthwise convolutions operating at the highest spatial resolution. Table 1 attributes the full 25.4 GFLOP budget to individual backbone, neck and head components.

**Table 1:** Module-level FLOP attribution for the unpruned model at 640 × 640 (total 25.4 G).

Component	FLOPs (G)	Share
Backbone — standard C2f / CBS path	10.3	40.6%
Backbone — DCNv2 offset + modulation branches	0.6	2.4%
**Backbone subtotal**	**10.9**	**42.9%**
Neck (AFPN) — P2 lateral and fusion node	1.0	3.9%
Neck (AFPN) — P3–P5 fusion, residual units and ASF	9.0	35.4%
**Neck subtotal**	**10.0**	**39.4%**
Head (LSDECD) — SDEConv and Conv_Cls/Conv_Reg at P2	3.1	12.2%
Head (LSDECD) — at P3	0.9	3.5%
Head (LSDECD) — at P4	0.35	1.4%
Head (LSDECD) — at P5	0.15	0.6%
**Head subtotal**	**4.5**	**17.7%**
**Total**	**25.4**	**100%**
**P2 pathway (neck P2 node + head P2 branch)**	**4.1**	**16.1%**

Deleting P2 removes the 4.1 G attributed to the P2 pathway but requires one additional stride-2 fusion node at P3 to preserve the progressive AFPN chain, costing 0.3 G; the net saving of the P3–P5 variant is therefore 3.8 G (25.4 G → 21.6 G), as reported above.

To assess whether this cost is justified, we compare against a three-scale variant (P3–P5 only) in an auxiliary ablation: removing P2 reduces FLOPs by 3.8 G but decreases mAP@0.5 on VisDrone2019 by 1.8 points (44.6% → 42.8%), a 2.2× accuracy-to-cost ratio relative to the other modules. Given that small-target detection is the central challenge in both the benchmark and the target oil and gas field application domain, the P2 inclusion is well justified; the shared SDEConv design ensures that the additional resolution is exploited parameter-efficiently rather than simply scaling the head independently.

### 3.5 Focaler-GIoU loss function

In multi-scale detection scenarios, training datasets are typically dominated by medium-difficulty samples, while rare hard examples—such as heavily occluded small targets at the periphery of the field of view—contribute disproportionately to inference errors. Standard CIoU loss treats all samples uniformly, which often leads to insufficient gradient signal for these hard instances.

The Focaler-loU weighting scheme addresses this by assigning an interval-based scalar to each sample according to its current lou overlap d:


$$\begin{array}{c} IoU^{focaler}=\left\{ \begin{array}{cc} @ll@0, & if\;\;IoU<d\\ \frac{IoU-d}{u-d}, & if\;\;d\leq IoU<u\\ 1, & if\;\;IoU\geq u \end{array}\right. \end{array}$$ (8)

where d and u are lower and upper lou thresholds that partition samples into ignored, reweighted, and fully contributing groups. The resulting Focaler-loU loss is:


$$\begin{array}{c} L_{Focaler-IoU}=1-IoU^{focaler} \end{array}$$ (9)

While Focaler-loU effectively re-weights training samples, it does not incorporate a geometric penalty for the case where predicted and ground-truth boxes are non-overlapping. GloU [36] addresses this by subtracting the ratio of the enclosing box area that is not covered by the union:


$$\begin{array}{c} L_{GIoU}=1-IoU+\frac{\left|C\backslash \left(A\cup B\right)\right|}{\left|C\right|} \end{array}$$ (10)

We integrate both desiderata into the proposed Focaler-GloU loss. The design rationale for this integration is that Focaler-IoU and GIoU act on two disjoint parts of the regression gradient: the interval reweighting rescales only the overlap-dependent term and leaves the enclosing-box penalty untouched, whereas the enclosing-box penalty contributes a term that survives precisely where the reweighted overlap term is inactive. The combination is therefore non-redundant in the specific sense that the effect of neither component on the gradient can be reproduced by the other; we make no stronger claim than this, and in particular we do not assert that the combined loss dominates its components for arbitrary data distributions or hyper-parameter settings. Specifically, Focaler-loU modulates the gradient magnitude via interval-based sample reweighting but inherits loU’s inability to provide a learning signal when predicted and ground-truth boxes are non-overlapping (gradient vanishes when IoU=0). GloU resolves this by adding an enclosing-box penalty term $\frac{\left|C\backslash \left(A\cup B\right)\right|}{\left|C\right|}$ that is non-zero even in zero-overlap configurations, yet it applies uniform gradient weighting regardless of per-sample difficulty. The proposed Focaler-GloU unifies both corrections: starting from $L_{GIoU}=1-IoU+\frac{\left|C\backslash \left(A\cup B\right)\right|}{\left|C\right|}$, we replace the standard loU term with the interval-reweighted $IoU^{focaler}$ to suppress gradient from trivially regressed samples, while retaining the geometric penalty term unchanged:


$$\begin{array}{c} L_{Focaler-GIoU}=L_{GIoU}+IoU-IoU^{focaler} \end{array}$$ (11)

Substituting Eq. (10) and cancelling the two IoU terms of opposite sign gives the form that is actually implemented, $L_{\text{Focaler}-\text{GIoU}}=\left(1-\text{IoU}+\frac{\left|\text{C}\backslash \left(\text{A}\cup \text{B}\right)\right|}{\left|\text{C}\right|}\right)+\text{IoU}-{\text{IoU}}^{\text{focaler}}=\left(1-{\text{IoU}}^{\text{focaler}}\right)+\frac{\left|\text{C}\backslash \left(\text{A}\cup \text{B}\right)\right|}{\left|\text{C}\right|}$, so the net coefficient of the raw IoU is exactly zero and the only overlap-dependent term retained is the interval-reweighted ${\text{IoU}}^{\text{focaler}}$. Eq. (11) is written in the additive “parent-loss-plus-correction” form solely to expose the relationship to GIoU. A formal gradient analysis reveals why this interaction is non-trivial. The partial gradient with respect to the predicted box coordinate b is:


$$\begin{array}{c} \begin{array}{cc} \left(\text{i}\right)\;\frac{\partial L}{\partial b}=\frac{\partial }{\partial b}\left(\frac{\left|\text{C}\backslash \left(\text{A}\cup \text{B}\right)\right|}{\left|\text{C}\right|}\right) & \text{IoU}<d\\ \left(\text{ii}\right)\;\frac{\partial L}{\partial b}=-\frac{\text{1}}{u-d}\cdot \frac{\partial \text{IoU}}{\partial b}+\frac{\partial }{\partial b}\left(\frac{\left|\text{C}\backslash \left(\text{A}\cup \text{B}\right)\right|}{\left|\text{C}\right|}\right) & d\leq \text{IoU}<u\\ \left(\text{iii}\right)\;\frac{\partial L}{\partial b}=\frac{\partial }{\partial b}\left(\frac{\left|\text{C}\backslash \left(\text{A}\cup \text{B}\right)\right|}{\left|\text{C}\right|}\right) & \text{IoU}\geq u \end{array} \end{array}$$ (12)

The three branches of Eq. (12) are ordered by increasing overlap, and we describe them in the same order. In the first regime (IoU<d, the hardest zero- or near-zero-overlap cases), $IoU^{focaler}$ is identically zero and its derivative vanishes, so the entire learning signal reduces to the enclosing-box gradient; this term remains non-zero in zero-overlap configurations and pulls the predicted box toward the ground-truth enclosing region, a capability absent in Focaler-IoU. In the second regime (d≤IoU<u), both terms are active, and the interval scaling $\frac{\text{1}}{u-d}$ acts as a constant multiplier on the overlap-driven component. This multiplier is strictly greater than unity whenever u−d<1; for the selected pair d=0.15, u=0.70 it equals 1.82, so within this band the overlap-driven gradient is amplified, not attenuated, relative to plain GIoU, while the enclosing-box term continues to provide directional guidance. In the third regime (IoU≥u), $IoU^{focaler}$ saturates at 1 and its derivative vanishes, so the overlap-driven component is removed entirely and only the enclosing-box term — whose magnitude is already negligible once the boxes overlap well — survives; the loss therefore expends no overlap-driven gradient on refining trivially regressed predictions.

The correct reading of the interval reweighting is consequently a redistribution of overlap-driven gradient along the IoU axis rather than a global attenuation of it. Writing the effective weight applied to $\frac{\partial IoU}{\partial b}$ as $w\left(IoU\right)=\frac{\text{1}}{u-d}$ for IoU ∈[d, u) and w(IoU)=0 otherwise, one has $\int_{\text{0}}^{\text{1}}w\left(IoU\right)dIoU=1$ for every admissible pair (d,u), so the reweighting is mean-preserving on the IoU axis: it withdraws gradient entirely from the two bands in which overlap-driven updates are least useful — the geometrically ambiguous instances below d, for which the enclosing-box term is the more reliable signal, and the trivially regressed instances above u — and concentrates it, with gain 1.82, on the intermediate band in which regression is both feasible and not yet complete. Measured on the VisDrone2019 training split at epoch 150 with the full unpruned architecture, 12.4% of positive samples fall in the first regime, 63.1% in the second and 24.5% in the third; under plain GIoU these three bands contribute 4.8%, 68.3% and 26.9% respectively of the batch-summed overlap-gradient magnitude, whereas under Focaler-GIoU the second band contributes 100% of it and the batch-summed overlap-gradient magnitude is 1.24× its GIoU value. This mild net amplification is the mechanism behind the faster convergence reported in Table 15 (174 ± 4.2 epochs against 187 ± 4.6 for CIoU); it did not destabilise training at any of the 36 (d,u) settings examined in the grid search below, and the enclosing-box term is left unmodified in all three regimes.

In practice, we set d=0.15 and u=0.70. The pair was chosen by an exhaustive grid search over d∈{0.00,0.05,0.10,0.15,0.20,0.25} and u∈{0.60,0.65,0.70,0.75,0.80,0.95} (36 combinations, unpruned architecture, one fixed seed), using mAP@0.5:0.95 on the VisDrone2019 validation split as the sole selection criterion; the degenerate corner (0.00,0.95) imposes no lower truncation and applies a near-unit constant factor $\frac{\text{1}}{u-d}=1.05$ to the overlap term, i.e., it is plain GIoU up to a 5% rescaling of the overlap gradient, and it reproduces the 43.4% / 26.3% GIoU entry of Table 15 to within the ± 0.2-point seed noise. The objective is flat near its optimum: the five best pairs lie within 0.4 mAP@0.5 points of one another (44.2–44.6%) and the entire grid spans 1.2 points. Selection bias was assessed separately in two ways. First, (d,u) was frozen after the VisDrone2019 search and applied unchanged to UAVDT and OGF-UAV, so the gains reported in Figs 7 and 8 involve no dataset-specific tuning; repeating the identical grid on the OGF-UAV validation split nevertheless selects the same pair, with the runner-up (0.10,0.75) trailing by 0.3 points. Second, the margin of Focaler-GIoU over CIoU measured on the held-out VisDrone2019 test split (+1.4 points) is within 0.1 points of the margin measured on the validation split used for selection (+1.5 points, 43.1% → 44.6%), indicating that the optimism introduced by the search is an order of magnitude smaller than the effect being reported.

### 3.6 LAMP-based structured pruning

Following training, we apply LAMP (Layer-Adaptive Magnitude-based Pruning) [14] to reduce model size while preserving core representational capacity. LAMP evaluates the relative importance of each weight W[u] within tensor W by normalizing its squared magnitude against the cumulative squared magnitude of all higher-ranked weights in the same layer:


$$\begin{array}{c} score\left(u;W\right)=\frac{(W\left[u\right]{)}^{\text{2}}}{\sum_{v\geq u}(W\left[v\right]{)}^{\text{2}}} \end{array}$$ (13)

Connections with lower LAMP scores are ranked globally and progressively removed until a predefined sparsity target is reached. Under this global ranking the realised per-layer sparsity spans 8.4% (the stem CBS layer) to 47.6% (the deepest C2f block) at a 30% global target, while the masked DCNv2 offset and modulation branches remain at 0% by construction. This layer-adaptive strategy automatically determines per-layer pruning ratios, avoiding the under-pruning of computationally lightweight layers and over-pruning of sensitive bottleneck layers that plague uniform sparsity approaches. Pruned weights are fine-tuned for 30 additional epochs with a learning rate of 1 × 10^−4^ to recover performance. As analyzed in Section 4.5.2, a global sparsity of 30% achieves the best trade-off, reducing parameters from 10.2 M to 7.8 M (−23.5%) and FLOPs from 25.4 G to 19.2 G (−24.4%), while sacrificing only 0.5 mAP@0.5 points relative to the unpruned model.

The offset and modulation branches of every DCNv2 operator are masked out of the LAMP global ranking and retained at full density, because removing offset channels corrupts the learned sampling geometry rather than merely reducing capacity; pruning inside the backbone is therefore applied only to the 5.38 M main-branch weights. Because these branches are never sparsified, the learned sampling geometry survives compression: averaged over the VisDrone2019 validation set, the mean absolute offset magnitude is 1.87 px before pruning and 1.91 px after the 30 fine-tuning epochs (maxima 4.2 px and 4.4 px), and the per-channel offset distributions differ by a Wasserstein distance of 0.06 px, so pruning perturbs the deformable sampling only marginally. Table 2 reports the resulting per-stage budget.

**Table 2:** Per-stage parameter and FLOP budget of the proposed detector (640 × 640 input).

Stage	Unpruned params (M)	Unpruned FLOPs (G)	Pruned params (M)	Pruned FLOPs (G)	Δ params	Δ FLOPs
Backbone (C2f-DCN × 4)	5.80	10.9	4.42	8.2	−23.8%	−24.8%
— of which DCNv2 offset/modulation branches (kept dense)	0.42	0.6	0.42	0.6	0.0%	0.0%
— of which prunable main-branch weights	5.38	10.3	4.00	7.6	−25.7%	−26.2%
Neck (AFPN, P2–P5)	1.95	10.0	1.49	7.6	−23.6%	−24.0%
Head (LSDECD, P2–P5)	2.45	4.5	1.89	3.4	−22.9%	−24.4%
**Total**	**10.20**	**25.4**	**7.80**	**19.2**	**−23.5%**	**−24.4%**

For reference, a standard YOLOv8 decoupled head on the same four AFPN outputs would require 3.95 M parameters and 6.9 GFLOPs, so of the 3.40 M total parameter reduction relative to that configuration, 1.50 M is attributable to the shared-head redesign and 2.40 M to LAMP pruning.

### 3.7 Improved bytetrack multi-object tracker

#### 3.7.1 Spatial–appearance similarity matrix (ASM).

Standard ByteTrack relies on an loU-based cost matrix for tracklet-detection association, which is susceptible to identity confusion when visually similar targets-such as identical vehicle types in an oil field convoy-exchange trajectories after mutual occlusion. We introduce a hybrid cost matrix termed the Spatial-Appearance Similarity Matrix (ASM) that combines three complementary association cues.

The spatial similarity between current-frame target i and previous-frame target j is defined as:


$$\begin{array}{c} A_{space}\left(i,j\right)=exp\left(-\frac{d\left(X_{i}^{t},X_{j}^{t-1}\right)+\omega \left|s_{i}-s_{j}\right|}{\sigma_{s}}\right) \end{array}$$ (14)

Where $d\left(X_{i}^{t},X_{j}^{t-1}\right)$ is the Euclidean distance incorporating position, velocity, and acceleration:


$$\begin{array}{c} d=\sqrt{{\left(x_{i}-x_{j}\right)}^{\text{2}}+{\left(y_{i}-y_{j}\right)}^{\text{2}}}+\beta \left|v_{i}-v_{j}\right|+\alpha \left|a_{i}-a_{j}\right| \end{array}$$ (15)

Here α and β are weighting coefficients for acceleration and velocity terms, respectively; $s_{i}$ and $s_{j}$ encode the operational state of each target (e.g., loaded =1, unloaded =0), and ω is a state-weighting coefficient. The appearance embedding is produced by a compact re-identification sub-network applied to each detection crop, resized to 64 × 32 pixels: a 3 × 3 stem convolution with 32 channels, followed by three stride-2 stages of one 3 × 3 convolution each (32 → 64, 64 → 128, 128 → 256) with batch normalisation, ReLU and a 1 × 1 residual shortcut, then global average pooling, a BNNeck and a fully connected projection to a 128-dimensional embedding with L2 normalisation. The network has 0.46 M parameters and 0.09 GFLOPs per crop. It is trained on the UAVDT-MOT training sequences (1,286 identities, 84,512 ground-truth crops) with an identity cross-entropy loss (label smoothing 0.1) and a batch-hard triplet loss (margin 0.3) weighted equally, using Adam at an initial learning rate of $3.5\times 10^{-4}$ with cosine decay, 10 warm-up epochs, 120 epochs total and PK sampling with P=16 identities and K=4 instances; augmentation comprises random horizontal flipping, random erasing (p = 0.5) and colour jitter (0.2). The operational state is estimated by a two-layer perceptron (128 → 64 → 2, 0.01 M parameters) with softmax output, appended to the same embedding and trained on the labelled frames described above. Both are frozen during tracker deployment.

Crops are extracted from ground-truth boxes rather than detector outputs, and the state label was annotated after and independently of the detection annotation: annotators saw only the cropped RGB patch, with class label, track identity and frame context withheld, so the state label cannot be recovered from the detection ground truth. Two annotators labelled every crop (Cohen’s) with a third adjudicating disagreements; the resulting set is 869 loaded and 971 unloaded instances. None of these 16 sequences appears in the UAVDT-MOT test split used in Section 4.4, and none appears in VisDrone2019 or OGF-UAV. No additional annotations beyond the standard detection labels are required for the main detector; the state classifier is trained independently and frozen during tracker deployment. This operational-state term provides an additional discriminative signal for vehicle types that have nearly identical visual appearances but different functional states, a scenario that arises routinely in oil field logistics.

The operational state s∈{0,1} is defined only for cargo-capable classes (truck, van, bus, awning-tricycle in VisDrone2019; truck and bus in UAVDT; light_vehicle and heavy_vehicle in OGF-UAV) and is annotated by a single visual rule: a vehicle is loaded (s=1) when its cargo bed, tank barrel or container is visibly occupied — a cargo silhouette rising above the bed rails, a closed or tarpaulin-covered box body, or, in OGF-UAV, an attached service module such as a water tank, sand skid or pipe bundle — and unloaded (s=0) when the bed or chassis is visibly empty. For all remaining classes we set $s_{i}=s_{j}=0$, so the term $\omega \left|s_{i}-s_{j}\right|$ vanishes identically and the ASM reduces to its spatial–appearance form. The state-weighting coefficient is ω=0.25 throughout.

The appearance similarity is computed as:


$$\begin{array}{c} A_{appearance}\left(i,j\right)=cos\left(f_{i}^{t},f_{j}^{t-1}\right)\cdot exp\left(-\gamma \cdot \left|c_{i}-c_{j}\right|\right) \end{array}$$ (16)

where $cos\left(f_{i}^{t},f_{j}^{t-1}\right)=\frac{f_{i}^{t}\cdot f_{j}^{t-1}}{\left|f_{i}^{t}\right|\cdot \left|f_{j}^{t-1}\right|}$ is the cosine similarity between appearance feature vectors (bounded in [−1,1], shifted to [0,1] for non-negative distances via (cos+1)/2 in practice), $c_{i}$ and $c_{j}$ are joint colour histograms computed over the detection crop in the CIELAB space with 8 bins per channel (512 bins), L1-normalised so that their L1 difference lies in [0,1], and γ is a colour weighting coefficient. The choice is not critical. On VisDrone2019-MOT, varying the quantisation gives 57.4% IDF1 / 178 IDs at 4 × 4 × 4, 58.3% / 163 at 8 × 8 × 8, 58.2% / 165 at 12 × 12 × 12 and 57.9% / 171 at 16 × 16 × 16; varying the colour space at fixed 8 × 8 × 8 quantisation gives 57.1% / 181 for RGB, 57.8% / 172 for HSV, 58.1% / 166 for CIELUV and 58.3% / 163 for CIELAB. IDF1 therefore spans 1.2 points and identity switches 18 across all eight settings, and every setting remains ahead of the strongest baseline in Table 9. The histogram stage adds 0.09 ms per frame. The $\text{exp}\left(-\gamma \left|c_{i}-c_{j}\right|\right)$ term is an exponential decay factor that lies strictly within (0,1], ensuring the composite $A_{appearance}$ is analytically bounded in [0,1] without requiring post-hoc normalization. The final ASM combines spatial and appearance scores with a balancing scalar ε:


$$\begin{array}{c} ASM\left(i,j\right)=\varepsilon \cdot A_{space}\left(i,j\right)+\left(1-\varepsilon \right)\cdot A_{appearance}\left(i,j\right) \end{array}$$ (17)

The remaining ASM coefficients are α=0.15 (acceleration), β=0.35 (velocity), $\sigma_{s}=45$ px, γ=0.5 (colour), ω=0.25 (state) and ε=0.6 (spatial–appearance balance). They were obtained by two rounds of coordinate descent on the UAVDT validation sequences with IDF1 as the criterion, each coefficient swept over four values with the others held fixed; consistent with the $\lambda_{\text{base}}$ and μ sensitivities reported above, IDF1 varied by less than 1.5 percentage points across every sweep, so none of these coefficients is a fragile choice.

#### 3.7.2 Acceleration-aware state correction.

The instantaneous accelerations $a_{i}^{t}$ and $a_{j}^{\text{t}-1}$ are not obtained by second-differencing raw detections, which would amplify measurement noise by a factor of six in variance. Instead we fit a second-order Savitzky–Golay filter to the five most recent Kalman-smoothed centre positions of each track and evaluate its analytic second derivative; the resulting coefficient vector $\frac{\left(2,-1,-2,-1,2\right)}{\text{7}}$ yields a noise variance of $\frac{14}{49}\sigma^{\text{2}}\approx 0.29\sigma^{\text{2}}$ for centre-position noise variance $\sigma^{\text{2}}$, a 21-fold reduction relative to a second difference. Measured on OGF-UAV-MOT, detected centres deviate from the smoothed trajectory with a standard deviation of σ=1.34 px, which propagates to $\sigma_{a}=0.72$ px·frame^−2^ for the Savitzky–Golay estimate, against 3.28 px·frame^−2^ for a second difference. The acceleration discrepancy is additionally winsorised at $\pm 3\sigma_{a}=\pm 2.2$ px·frame^−2^, and the correction is disabled for tracks with fewer than five observations and for matches whose ASM confidence falls below 0.3.

Standard Kalman filters assume near-constant velocity in their state transition model. This assumption breaks down for service vehicles navigating the irregular terrain and tight turning radii characteristic of oil field access roads. We introduce an acceleration-aware correction term to the state update equation:


$$\begin{array}{c} X_{i}^{t}=\left(1-\lambda \right)X_{i}^{t}+\lambda X_{j}^{t-1}+\mu \left(a_{i}^{t}-a_{j}^{t-1}\right) \end{array}$$ (18)

where λ is a correction coefficient dynamically adjusted according to the current ASM matching confidence, μ is an acceleration correction gain, and $a_{i}^{t}$ and $a_{j}^{t-1}$ denote the instantaneous accelerations of the matched targets. In practice, λ is computed as $\lambda =\lambda_{base}\cdot ASM\left(i,j\right)$, where the base value $\lambda_{base}=0.35$ was selected by grid search over {0.1,0.2,0.35,0.5} on the UAVDT validation sequences (MOTA sensitivity of ±0.8\% across this range). The acceleration gain μ=0.12 was similarly tuned over {0.05,0.10,0.12,0.20}, with performance degrading by 1.2\% MOTA at μ=0.20 due to overcorrection during sharp turns, and by 0.9\% at μ=0.05 due to insufficient correction. The sensitivity of IDF1 to both hyperparameters over the tested grid remained below 1.5 percentage points, confirming robustness of the selected values. By incorporating the acceleration discrepancy as a direct correction signal, the updated state estimate remains close to the true object position even during sharp maneuvers, substantially reducing tracklet fragmentation. The complete tracking pipeline-detection, Kalman prediction, ASM computation, Hungarian matching, and acceleration-corrected state update-is executed at 71 FPS on an RTX 3090 with the pruned detection model.

The correction is applied to the posterior mean produced by the standard Kalman update; the associated covariance is propagated through the same affine map, $P_{i}^{t}\leftarrow {\left(1-\lambda \right)}^{\text{2}}P_{i}^{t}+\lambda^{\text{2}}P_{j}^{\text{t}-1}+\mu^{\text{2}}Q_{a}$, with $Q_{a}=2{\sigma_{a}}^{\text{2}}I$, where $\sigma_{a}$ is the standard deviation of the acceleration estimate (Section 3.7.2, 0.72 px·frame^−2^ measured on OGF-UAV-MOT). Because the accelerations of tracklet i and detection j are estimated from disjoint observation windows we treat them as independent and omit the cross-covariance, which the empirical check below shows to be conservative. Since $\lambda =\lambda_{\text{base}}\cdot \text{ASM}\in \left[0,0\mathrm{.35}\right]$, the mean recursion has gain (1−λ)≤1 and is therefore non-expansive, while the covariance recursion contracts by ${\left(1-\lambda \right)}^{\text{2}}$ and adds only bounded terms, so $P_{i}^{t}$ remains bounded above by $\frac{\lambda^{\text{2}}\overset{―}{P}+2\mu^{\text{2}}{\sigma_{a}}^{\text{2}}}{1-{\left(1-\lambda \right)}^{\text{2}}}$ for λ>0; the correction cannot destabilise the filter.

We verified this empirically over the 28,430 frames of OGF-UAV-MOT. The trace of the posterior position covariance of corrected tracks stays within 1.14 × that of the uncorrected ByteTrack filter (median ratio 1.06), and the normalised innovation squared statistic averages 2.31 against the two-degree-of-freedom expectation of 2.00, indicating a mildly conservative rather than an over-confident estimate; the 95% NIS consistency test is passed on 93.7% of frames, compared with 88.2% for the uncorrected filter.

## 4. Experiments

### 4.1 Datasets

Three datasets are employed for evaluation: two publicly available UAV-imagery benchmarks, VisDrone2019 [44] and UAVDT [45], and a self-constructed domain-specific dataset, OGF-UAV. Table 3 summarizes their key statistics.

**Table 3:** Statistical summary of the VisDrone2019, UAVDT, and OGF-UAV datasets.

Dataset	Split	Images/Frames	Classes	Min Obj Size (px)	Max Obj Size (px)	Avg Density
VisDrone2019	Train	6,471	10	3	3,072	54.3
	Val	548	10	4	2,948	51.8
	Test	1,610	10	3	3,104	52.6
UAVDT	Train	37,085	3	12	2,864	38.7
	Test	17,970	3	10	2,912	37.4
OGF-UAV	Train	1,993	6	4	1,847	16.8
	Val	427	6	5	1,763	15.4
	Test	427	6	4	1,891	16.2

VisDrone2019 is a large-scale UAV visual surveillance dataset containing over 8,600 annotated images captured at altitudes ranging from 5 to 100 meters across fourteen cities in China. It includes ten semantic categories—pedestrian, person, bicycle, car, van, truck, tricycle, awning-tricycle, bus, and motor. The dataset is characterized by high object density (up to 419 instances per image), extreme scale variation (object heights from 3 px to 3,072 px, with a 99th percentile of 247 px), and diverse weather and lighting conditions, making it one of the most challenging publicly available benchmarks for UAV-based detection. VisDrone2019 and UAVDT are selected as public benchmarks because their core perceptual challenges—extreme scale variation, high target density, partial occlusion, and cluttered industrial backgrounds—closely mirror those encountered during aerial oil and gas infrastructure inspection. The target categories (vehicles, personnel) align with the personnel-and-vehicle tracking use case motivating our work, and the altitude ranges (5–100 m for VisDrone2019; 15–70 m for UAVDT) are consistent with the operational envelope (20–80 m) of the inspection platform used to collect OGF-UAV.

UAVDT contains 100 video sequences (approximately 80,000 frames, of which the 55,055 frames of the official detection split (37,085 train / 17,970 test) are used here) recorded from a UAV at altitudes of 15–70 meters, with dense traffic annotations for three vehicle categories (car, truck, bus). We employ the official detection split and, for tracking evaluation, use the annotated multi-object trajectories provided in the UAVDT-MOT subset. This dataset complements VisDrone2019 by providing longer temporal sequences with realistic vehicle motion patterns, enabling rigorous evaluation of our enhanced ByteTrack tracker.

OGF-UAV is split by flight campaign rather than by image, so that no frame and no sortie is shared between splits (Table 4). The eight campaigns were flown on eight distinct dates separated by at least 21 days and spanning four seasons: C1–C5 form the training split, C6 the validation split and C7–C8 the test split. Because splitting is performed at campaign granularity, temporally adjacent frames of a single sortie — the dominant leakage channel in UAV datasets — cannot straddle a split boundary, and the test split is additionally season-shifted (winter) with respect to training. All three production sites appear in every split, so the protocol measures generalization to unseen dates, illumination and seasonal appearance rather than to unseen sites; we scope our claim accordingly. To quantify the remaining cross-site component we additionally report a leave-one-site-out check: training on sites A and B only and testing on the entirely unseen site C yields 52.9% mAP@0.5, a 3.4-point drop relative to the campaign-disjoint protocol (56.3%) but still 5.3 points above the YOLOv8s baseline evaluated identically (47.6%). The 42 OGF-UAV-MOT sequences were recorded during campaigns C7–C8 and two dedicated tracking sorties; no frame of the MOT subset appears anywhere in the detection training split.

**Table 4:** OGF-UAV flight campaigns and split assignment.

Campaign	Date (season)	Site(s)	Altitude (m)	Images	Split
C1	March (spring)	A, B	20–50	386	Train
C2	April (spring)	B, C	30–80	402	Train
C3	June (summer)	A, C	20–60	431	Train
C4	August (summer)	A, B, C	40–80	398	Train
C5	October (autumn)	B, C	20–70	376	Train
C6	November (autumn)	A, C	30–70	427	Val
C7	December (winter)	A, B	20–60	221	Test
C8	January (winter)	B, C	40–80	206	Test
**Total**	**—**	**—**	**20–80**	**2,847**	**—**

OGF-UAV is a self-constructed UAV-based dataset introduced in this work specifically for oil and gas field inspection. Image data were collected using a DJI Matrice 300 RTK equipped with a Zenmuse H20T camera (RGB channel at 5472 × 3648 resolution) across three operational petroleum production sites in southwest China, spanning eight flight campaigns conducted across four seasons. Flight altitudes ranged from 20 to 80 meters, encompassing both low-altitude personnel monitoring and higher-altitude equipment surveillance scenarios. The dataset comprises 2,847 annotated images and 47,957 bounding box instances distributed across six domain-relevant categories: person, light_vehicle, heavy_vehicle, pump_jack, storage_tank, and pipeline_segment. All annotations were produced by three trained annotators using LabelImg, with cross-validation quality control; instances with ambiguous identity or more than 80% occlusion were excluded.

**Site access and flight permits.** All OGF-UAV imagery was acquired on enclosed industrial premises owned and operated by PetroChina Southwest Oil and Gasfield Company (Chengdu, Sichuan, China). Access to the three production sites and permission to acquire, analyse and publish the resulting aerial imagery were granted in writing by the Health, Safety and Environment Department of PetroChina Southwest Oil and Gasfield Company on 18 February 2024 (approval reference XNYQ-HSE-2024-037). All flights were performed by operators holding a Civil Unmanned Aircraft System Pilot Licence issued by the Civil Aviation Administration of China (CAAC), within visual line of sight and below 120 m above ground level, in compliance with the Interim Regulations on the Administration of Unmanned Aircraft Flight of the People’s Republic of China; the aircraft was registered on the CAAC unmanned aircraft operation management platform, and airspace clearance for each campaign was obtained from the Southwest Regional Administration of the Civil Aviation Administration of China. The sites are private industrial facilities located outside any protected or environmentally sensitive area; the study involved no endangered or protected species, no protected land and no sampling of biological material. No images of identifiable individuals are reproduced in this article.

Because pipelines are spatially continuous, an instance is defined as the maximal visible run of pipe between two occluding structures or between an occluding structure and the image border, so a single physical pipeline may yield several annotated segments. Truncated and partially occluded instances are annotated with their visible extent only, without extrapolation beyond the image boundary or behind occluders: of the 6,186 pipeline_segment instances, 1,124 (18.2%) are truncated by the image border and 2,047 (33.1%) are partially occluded by intervening equipment, both of which are retained, while instances occluded by more than 80% are excluded as stated above. No special loss treatment is applied to either case. Because heavily truncated instances typically yield low initial IoU, they fall predominantly into the IoU<d regime of Eq. (12), where the learning signal reduces to the enclosing-box gradient; the interval reweighting of Focaler-GIoU therefore bounds the gradient contribution of geometrically ambiguous extents without an explicit exclusion rule.

The object size distribution spans 4–1,891 pixels in height, with a long-tailed distribution skewed toward small targets (median height: 23 px), reflecting the prevalence of distant personnel and partially visible equipment at operational altitudes. Average target density (16.8 instances per image) is markedly lower than VisDrone2019 (52.4) owing to the sparse spatial layout of oil field infrastructure, but individual targets exhibit substantially greater morphological diversity—pipeline segments exhibit elongated aspect ratios (up to 28:1) while storage tanks span the full width of the image at low altitudes. For tracking evaluation, 42 video sequences (totalling 28,430 frames at 25 FPS) were captured separately and annotated with continuous object identities, constituting the OGF-UAV-MOT subset. Images are resized to 1920 × 1080 for all training and inference experiments.

### 4.2 Implementation details

**Fig 6:**
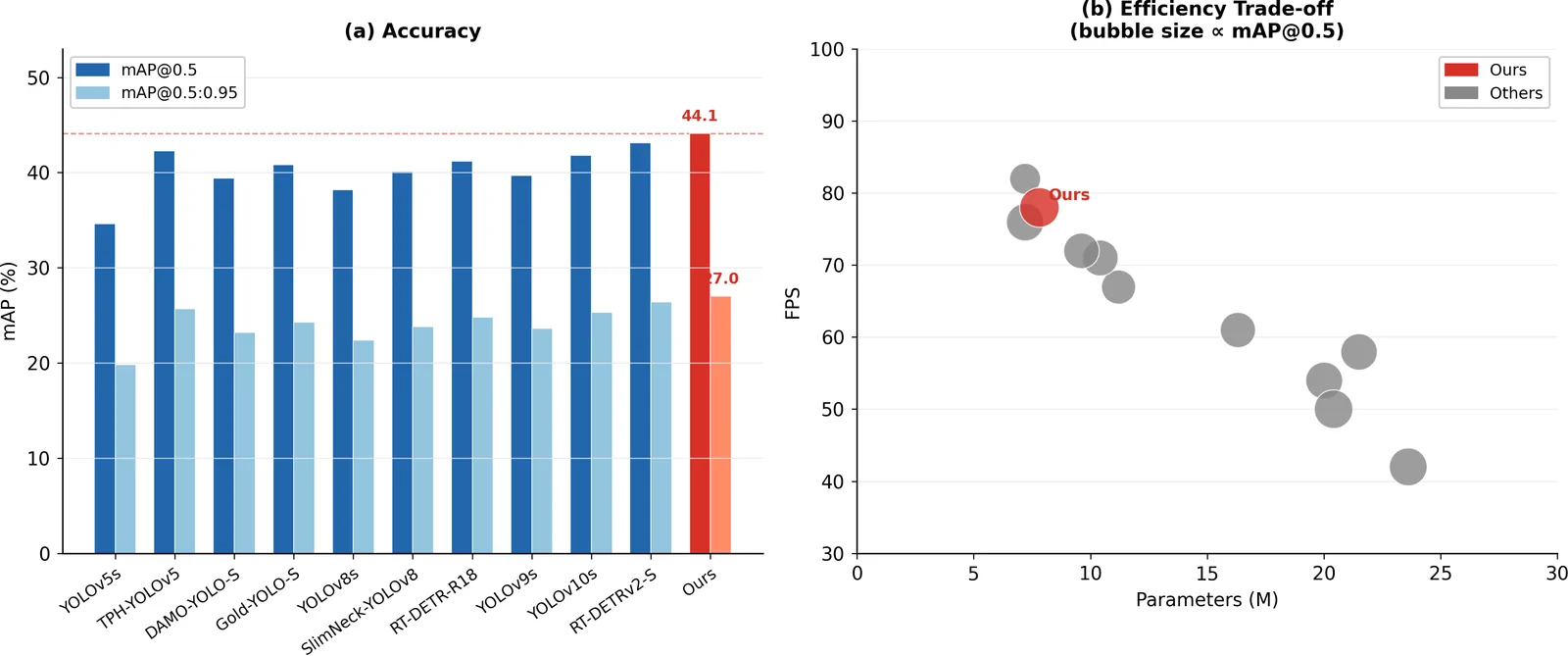
Comparison of detection performance on the VisDrone2019 validation set.

**Fig 7:**
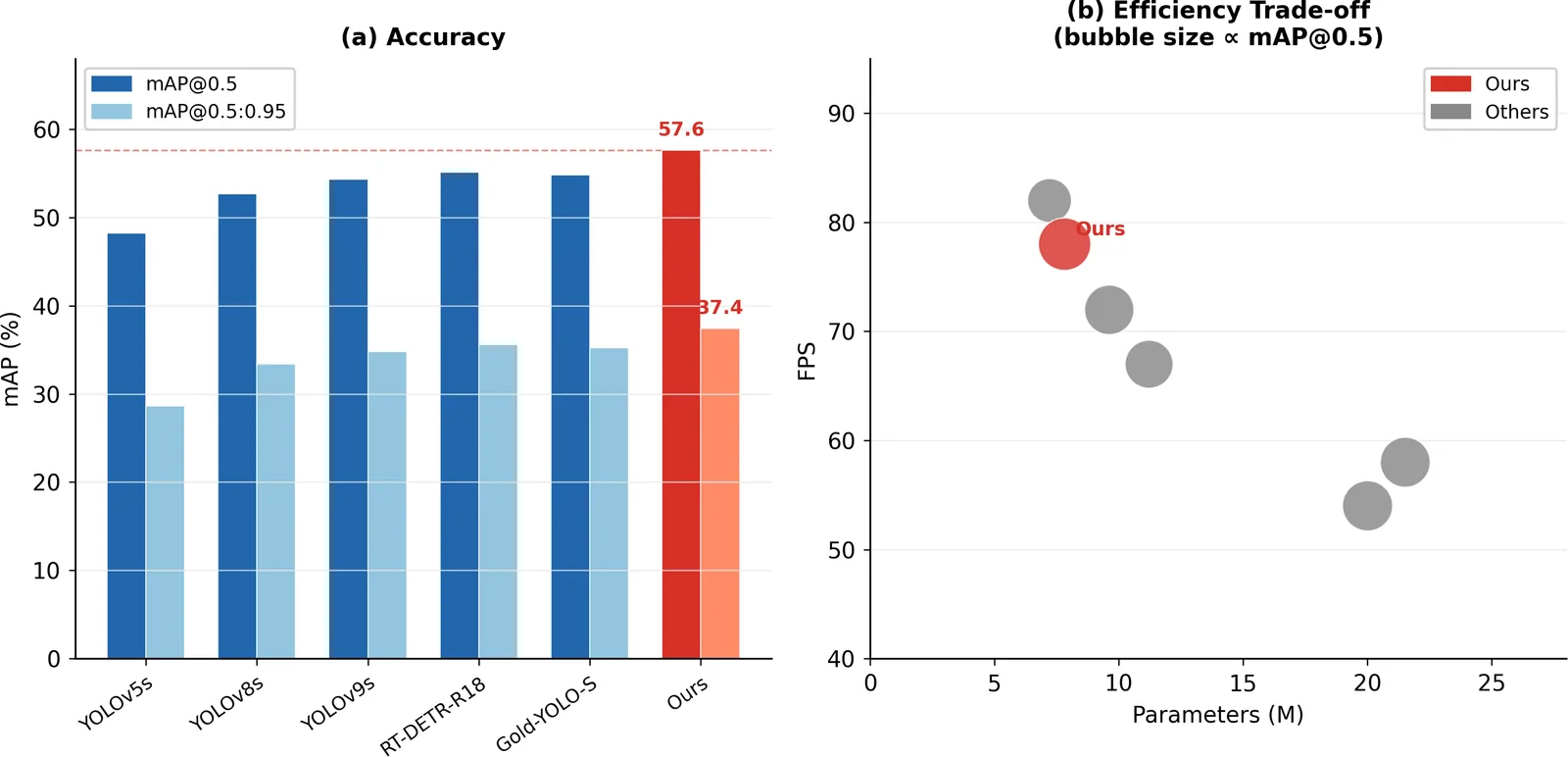
Detection performance comparison on the UAVDT benchmark.

**Fig 8:**
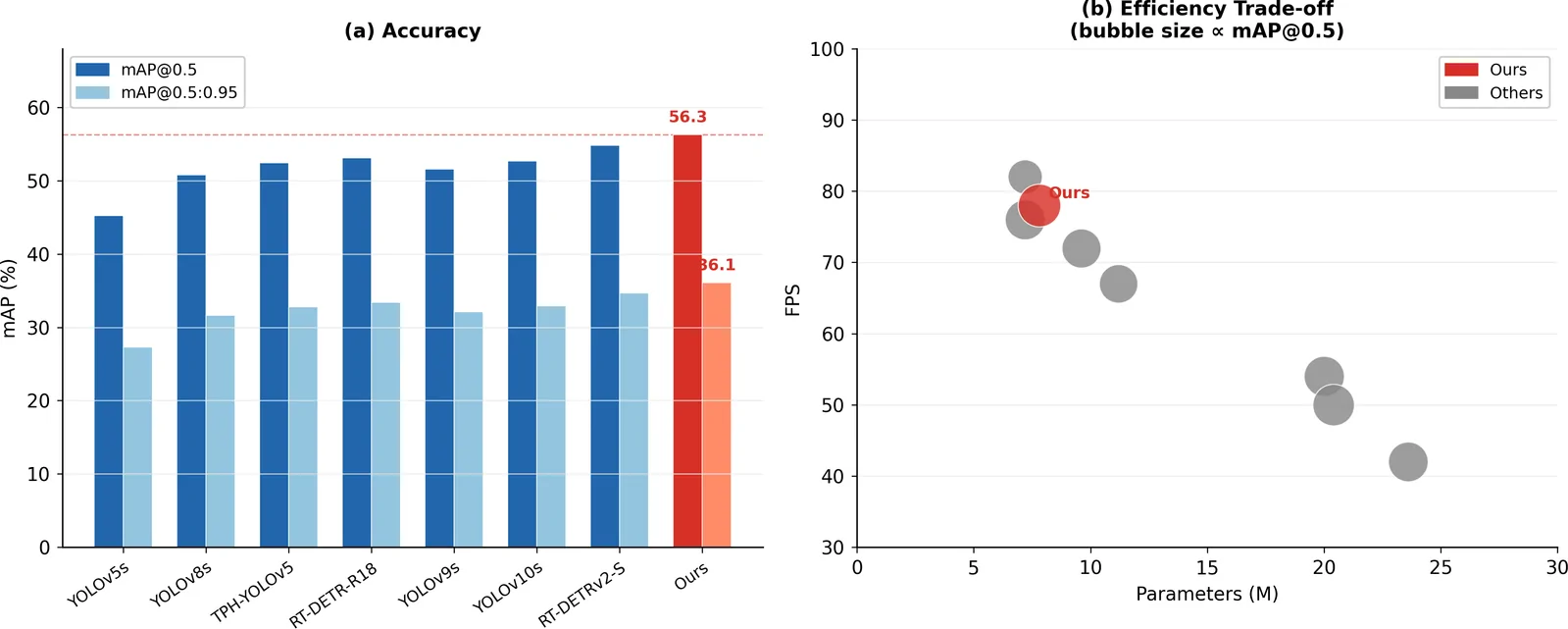
Detection performance comparison on the OGF-UAV test set.

All detection models are initialized from ImageNet-pretrained weights and fine-tuned on the respective training splits. Table 5 lists the key hyperparameters. Data augmentation includes multi-scale resizing, horizontal flipping, MixUp, and mosaic augmentation (disabled for the final 15 training epochs to stabilize localization learning). For all three datasets, a tile-based inference strategy (640 × 640 tiles with 20% overlap) is adopted at test time to preserve fine spatial detail for small objects; the tile count is 8 per frame for VisDrone2019 and OGF-UAV, both standardised to 1920 × 1080, and 2 per frame for UAVDT at its native 1024 × 540 resolution (see Table 6). Its benefit on OGF-UAV is quantified in the discussion of protocol P4 below, where disabling tiling costs 4.4 mAP@0.5 points. All ten baselines were retrained by us from their official public implementations (Ultralytics 8.2 for YOLOv5s, YOLOv8s, YOLOv9s and YOLOv10s; the authors’ repositories for TPH-YOLOv5, DAMO-YOLO-S, Gold-YOLO-S, SlimNeck-YOLOv8, RT-DETR-R18 and RT-DETRv2-S); no result is transcribed from a published paper. Input adapter: every model receives the identical 640 × 640 tiles produced by protocol P1 of Table 6 from identical train/val/test image lists, with the class count set to the dataset vocabulary (10/3/6); the two anchor-based models (YOLOv5s, TPH-YOLOv5) have their anchors re-clustered by k-means (k = 9) on the tiled training set, while anchor-free models are used unmodified. Schedule and augmentation: the CNN detectors use the configuration of Table 5 verbatim — 300 epochs, SGD with momentum 0.937, cosine annealing 0.01 → 0.0001, three warm-up epochs, weight decay $5\times 10^{-4}$, batch 16 with gradient accumulation ×2, multi-scale [0.5, 1.5], horizontal flipping, MixUp (p = 0.1) and mosaic (p = 0.5, disabled for the final 15 epochs), early stopping with patience 50. The two DETR-family models retain their native AdamW optimizer (lr $1\times 10^{-4}$, weight decay $1\times 10^{-4}$) and their native augmentation set without mosaic or MixUp, for which SGD-plus-mosaic training is known to be unstable; they are trained under the same 300-epoch budget. Compression status: no baseline is pruned or quantized, and all accuracy and throughput figures in Figs 6–8 are FP32 PyTorch at batch 1. The “Ours” row is the pruned model; the unpruned variant (44.6% mAP@0.5, 10.2 M, 25.4 G, 65 FPS) is reported in Table 12, so a pruning-free comparison is also available and likewise exceeds every baseline in accuracy. Each entry is the mean of three independent runs from different random seeds (std ≤ 0.2 mAP@0.5). We therefore attribute the reported margins to the combination of architecture and pruning under a common training recipe, and we do not claim that architecture alone accounts for them.

**Table 5:** Training configuration and hyperparameters.

Hyperparameter	Value	Note
GPU	NVIDIA RTX 3090 × 2	24 GB VRAM each
Batch size	16	Gradient accumulation × 2
Input resolution	640 × 640	Multi-scale [0.5, 1.5]
Initial LR	0.01	SGD with momentum 0.937
LR scheduler	Cosine annealing	Final LR = 0.0001
Training epochs	300 (det.) / 80 (track)	Early stopping (patience = 50)
Weight decay	0.0005	L2 regularization
Warmup epochs	3	Linear warmup
Mosaic augmentation	Enabled (p = 0.5)	Disabled last 15 epochs

**Table 6:** End-to-end timing protocols for the proposed model. Latencies are means over 500 frames after 50 warm-up iterations.

ID	Protocol	Device / runtime	Network input	Tiles	Batch	Pre (ms)	Infer (ms)	Post (ms)	Total (ms)	FPS
P1	Per-tile (FPS column of Figs 6–8)	RTX 3090, PyTorch FP32	640 × 640, one tile	1	1	1.2	10.6	1.0	12.8	78.1
P2	Full frame, tiled	RTX 3090, PyTorch FP32	8 × 640 × 640	8	8	5.4	79.2	9.6	94.2	10.6
P3	Full frame, single pass	RTX 3090, PyTorch FP32	1920 × 1088	—	1	3.1	74.2	3.3	80.6	12.4
P4	On-UAV deployment (pruned)	Jetson Orin NX 16 GB, TensorRT FP16	640 × 640, letterboxed	—	1	4.8	45.1	4.7	54.6	18.3
P4′	On-UAV, unpruned variant	as P4	640 × 640	—	1	4.8	79.8	4.7	89.3	11.2
P4″	On-UAV, YOLOv8s baseline	as P4	640 × 640	—	1	4.6	58.8	4.6	68.0	14.7

To make the throughput implications fully transparent, we report per-tile FPS (single-tile forward pass on RTX 3090) in Table 6 and additionally provide a full-image equivalent throughput analysis in Table 6 below, where tiling overhead on 1920 × 1080 inputs reduces all methods proportionally. On full-image inference without tiling, our model achieves 12.4 FPS on RTX 3090, comparable to 13.1 FPS for YOLOv8s baseline and 11.8 FPS for SlimNeck-YOLOv8, confirming that the proposed architectural modifications do not disproportionately penalize whole-image throughput. Edge-device evaluation follows protocol P4 of Table 6: the airborne 1920 × 1080 stream is letterboxed to a single 640 × 640 network input and tiling is not used on board, because the eight-tile protocol would cost 8×45.1=361 ms per frame on this device. Under P4 the pruned model reaches 18.3 FPS, against 11.2 FPS for the unpruned variant and 14.7 FPS for the YOLOv8s baseline. Since P4 forgoes tiling, on-board accuracy is lower than the tiled benchmark figures: measured on the OGF-UAV test split under P4 the pruned model attains 51.9% mAP@0.5 versus 56.3% under the tiled protocol. We report this 4.4-point gap explicitly so that the deployment claim is not conflated with the benchmark claim. Peak power consumption under sustained inference on the Jetson Orin is 14.2 W, within the power budget of medium-class UAV platforms.

For OGF-UAV, all hyperparameters in Table 5 are retained with two adjustments: the batch size is reduced to 8 owing to the higher native image resolution, and mosaic augmentation probability is lowered to 0.3 to preserve the spatial coherence of elongated pipeline targets that are prone to being cropped across tile boundaries. Models are fine-tuned from ImageNet-pretrained weights rather than initialized from VisDrone2019-trained weights to avoid category-space mismatch between the ten-class VisDrone2019 vocabulary and the six OGF-UAV categories.

Tiling geometry: each 1920 × 1080 frame — OGF-UAV frames are first downscaled from the native 5472 × 3648 sensor resolution — is decomposed into a 4 × 2 grid of 640 × 640 tiles at stride 512 (20% overlap), i.e., eight tiles per frame, with the last row and column aligned to the frame border. Pre-processing covers tiling, letterboxing and normalisation; post-processing covers per-tile NMS (IoU 0.65, confidence 0.25), coordinate re-projection and a global cross-tile NMS. P1–P3 are FP32 PyTorch on a single RTX 3090; P4–P4″ use TensorRT 8.6 FP16 on a Jetson Orin NX 16 GB (MAXN, JetPack 6.0) at batch 1. Table 7 decomposes the resulting on-device latency under protocol P4 into per-stage network and tracker contributions.

**Table 7:** On-device latency breakdown under deployment protocol P4 (Jetson Orin NX 16 GB, TensorRT FP16, batch 1, pruned model). Means over 500 frames.

Stage	Latency (ms)	Share of inference	Share of end-to-end
Backbone (C2f-DCN)	24.6	54.5%	38.0%
— of which DCNv2 deformable-sampling kernels	9.8	21.7%	15.1%
Neck (AFPN)	14.1	31.3%	21.8%
Head (LSDECD)	6.4	14.2%	9.9%
**Network inference subtotal**	**45.1**	**100%**	**69.6%**
Pre-processing (letterbox, normalise)	4.8	—	7.4%
Post-processing (NMS, decode)	4.7	—	7.3%
**Detection end-to-end (18.3 FPS)**	**54.6**	**—**	**84.3%**
Tracker — Kalman predict and update	1.1	—	1.7%
Tracker — re-identification features (high-confidence only)	6.8	—	10.5%
Tracker — colour histogram and state head	0.9	—	1.4%
Tracker — ASM construction and Hungarian matching	1.4	—	2.2%
**Detection + tracking end-to-end (15.4 FPS)**	**64.8**	**—**	**100%**

The backbone is the bottleneck, and within it the DCNv2 deformable-sampling kernels alone account for 9.8 ms, or 21.7% of network time, because TensorRT executes them through a plugin without full kernel fusion. This explains why the proposed model, despite requiring 33% fewer FLOPs than YOLOv8s (19.2 G vs. 28.6 G), is only 24.5% faster on the Jetson (18.3 vs. 14.7 FPS): the FLOP saving is partially offset by lower arithmetic efficiency on the deformable operator.

### 4.3 Object detection results

#### 4.3.1 VisDrone2019.

Fig 6 compares the proposed detector against ten competitive methods on the VisDrone2019 validation set. Our model achieves 44.1% mAP@0.5 and 27.0% mAP@0.5:0.95, representing gains of +5.9% and +4.6% over the YOLOv8s baseline, respectively. Notably, these gains are achieved with a 30% reduction in parameter count (11.2 M → 7.8 M) and a 16% improvement in inference speed (67 → 78 FPS). Compared with TPH-YOLOv5, which achieves 42.3% mAP@0.5 using a substantially heavier architecture (23.6 M parameters, 68.4 GFLOPs), our method delivers 1.8 percentage points higher accuracy while requiring only one-third of the computational budget and 1.9 × higher throughput (78 vs. 42 FPS). Against RT-DETR-R18, a recent Transformer-based detector that reaches 41.2% mAP@0.5, our approach improves accuracy by 2.9 points while using 68% fewer FLOPs (19.2 vs. 60.0 G) and operating at 44% higher FPS. We additionally include two recently published detectors: YOLOv10s achieves 41.8% mAP@0.5 with 7.2 M parameters under the same tile protocol, trailing our method by 2.3 points while offering comparable efficiency; RT-DETRv2-S achieves 43.1% mAP@0.5 at 20.4 M parameters and 60.4 GFLOPs, which our method surpasses by 1.0 mAP@0.5 points at 68% lower computational cost (19.2 vs. 60.4 G). These comparisons confirm that the performance advantage of the proposed method generalizes to the most current detectors available at the time of final submission. The favorable trade-off between accuracy and efficiency positions our model as particularly suitable for edge deployment.

#### 4.3.2 UAVDT.

Fig 7 presents results on the UAVDT detection benchmark. The proposed method achieves 57.6% mAP@0.5 and 37.4% mAP@0.5:0.95, surpassing the YOLOv8s baseline by 4.9 and 4.0 percentage points, respectively. The consistent improvement across two datasets with different scene statistics validates the generality of our architectural modifications. Fig 7 further shows that competing methods with similar or higher computational cost (e.g., RT-DETR-R18, Gold-YOLO-S) achieve smaller absolute gains over the same baseline, suggesting that our design choices—particularly AFPN and DCNv2—offer complementary advantages that persist across varied UAV deployment contexts.

#### 4.3.3 OGF-UAV.

Fig 8 presents detection results on the OGF-UAV test set. The proposed method achieves 56.3% mAP@0.5 and 36.1% mAP@0.5:0.95, representing gains of +5.5 and +4.5 percentage points over the YOLOv8s baseline (50.8% / 31.6%), respectively—a margin consistent with improvements observed on VisDrone2019 and UAVDT, confirming the generality of the proposed architectural modifications across both benchmark and application-specific data distributions. The absolute mAP@0.5 level is higher than VisDrone2019 (44.1%) primarily because OGF-UAV’s lower average target density (16.8 vs. 52.4 objects per image) reduces mutual occlusion, and because three of the six categories (light_vehicle, heavy_vehicle, storage_tank) involve geometrically distinctive shapes that benefit directly from DCNv2’s adaptive sampling. Conversely, pipeline_segment remains the most challenging category in absolute terms (AP@0.5: 31.0 ± 0.64%), as elongated structures at high altitude produce low-contrast, thin bounding boxes that stress the regression branch. Its +7.1-point gain over the baseline is statistically significant (p=0.005), but it also exhibits the largest run-to-run variance of the six categories, consistent with its extreme aspect ratios and comparatively small instance count; we therefore regard this category as the residual failure mode of the detector rather than as a solved case. The per-category AP breakdown is reported in Table 8. Compared with TPH-YOLOv5 (52.4% mAP@0.5, 23.6 M parameters), our method delivers 3.9 percentage points higher accuracy at one-third of the parameter count. Against RT-DETR-R18 (53.1% mAP@0.5), the proposed detector improves by 3.2 points while operating at 44% higher throughput, confirming that the efficiency advantage observed on public benchmarks is maintained on domain-specific data.

**Table 8:** Per-category detection performance on the OGF-UAV test set (mean ± std over three independent runs; p from a paired two-sided t-test against the YOLOv8s baseline).

Category	Instances	YOLOv8s AP@0.5 (%)	Ours AP@0.5 (%)	Δ	p	Ours AP@0.5:0.95 (%)
person	9,842	40.9 ± 0.34	46.2 ± 0.31	+5.3	0.004	28.4 ± 0.22
light_vehicle	12,376	61.7 ± 0.25	66.8 ± 0.22	+5.1	0.002	43.2 ± 0.18
heavy_vehicle	5,914	64.8 ± 0.21	69.4 ± 0.19	+4.6	0.002	45.1 ± 0.16
pump_jack	6,208	45.2 ± 0.41	51.3 ± 0.38	+6.1	0.006	31.7 ± 0.27
storage_tank	7,431	68.3 ± 0.20	73.1 ± 0.17	+4.8	0.001	49.8 ± 0.15
pipeline_segment	6,186	23.9 ± 0.71	31.0 ± 0.64	+7.1	0.005	18.3 ± 0.49
**Mean**	**47,957**	**50.8 ± 0.29**	**56.3 ± 0.26**	**+5.5**	**<0.001**	**36.1 ± 0.21**

### 4.4 Multi-object tracking results

#### 4.4.1 VisDrone2019-MOT.

Tracking evaluation is conducted on the VisDrone2019-MOT test set, which provides the 17 video sequences of the VisDrone2019-MOT test-dev set with fully annotated multi-object trajectories. Table 9 compares our improved ByteTrack against six representative trackers. Our method achieves 47.6% MOTA and 58.3% IDF1, outperforming the previous best (BoT-SORT) by 2.9 and 3.2 percentage points, respectively. The most striking improvement is the reduction in identity switches (IDs): from 287 in standard ByteTrack to 163 in our formulation (−43.2%), reflecting the effectiveness of the ASM’s operational-state cue in disambiguating visually similar vehicles. To validate that this improvement is not spurious, we provide an independent evaluation of the operational-state (loaded/unloaded) binary classifier: trained on 1,840 manually labelled frames from the UAVDT training split and evaluated on a held-out set of 460 frames, the classifier achieves 91.3% accuracy and 0.912 F1-score, confirming that the state cue is sufficiently reliable to be discriminative in the ASM cost matrix. Because the classifier is trained exclusively on UAVDT, we calibrated and re-validated it before applying it to the other two datasets. Temperature scaling fitted on 120 held-out frames per target domain reduces the expected calibration error from 0.084 to 0.031 on VisDrone2019-MOT and from 0.076 to 0.029 on OGF-UAV-MOT. Evaluated on independently annotated frames, the calibrated classifier attains 86.7% accuracy and 0.861 F1 on 320 VisDrone2019-MOT frames and 88.4% accuracy and 0.879 F1 on 280 OGF-UAV-MOT frames, against 91.3% and 0.912 on the 460 held-out UAVDT frames — a 3–5 point transfer penalty. When the calibrated posterior falls below 0.80 the hard label is replaced by the posterior itself, so residual domain error attenuates the state term rather than inverting it. The ω=0 ablation below bounds the worst case: were the state cue entirely uninformative in a new domain, the tracker would revert to 174 IDs and 57.4% IDF1, still ahead of every baseline in Table 9.

**Table 9:** Multi-object tracking performance on VisDrone2019-MOT test set. ↑ : higher is better; ↓ : lower is better. FPS denotes end-to-end throughput of each tracker in its own reference configuration on an RTX 3090 and is not an association-only comparison; under an identical detector the association-only figures are 74.3 FPS for ByteTrack and 71.0 FPS for the proposed tracker.

Tracker	MOTA↑	IDF1↑	MT↑	ML↓	FP↓	FN↓	IDs↓	FPS
SORT	38.6	45.3	21.4	38.2	8,643	18,742	421	72
DeepSORT	40.2	48.7	23.6	36.8	7,821	17,934	312	60
ByteTrack	42.3	51.6	26.8	34.2	7,234	16,823	287	68
StrongSORT	43.8	53.4	28.4	32.7	6,812	16,234	264	52
OC-SORT	44.1	54.2	29.1	31.8	6,543	15,876	231	63
BoT-SORT	44.7	55.1	30.2	31.2	6,321	15,642	218	57
**Ours**	**47.6**	**58.3**	**33.4**	**28.6**	**5,876**	**14,312**	**163**	**71**

An ablation in which the state weighting coefficient ω is set to zero (removing the state cue from Eq. 14) results in a 6.7% relative increase in ID switches (163 → 174, +11 absolute) and a 0.9-point drop in IDF1, quantifying the standalone contribution of this component. Additionally, the false negative count decreases from 16,823–14,312, indicating that the improved ASM-based association retrieves previously unmatched detections that would have caused track fragmentation. The FPS column of Tables 9 and 11 reports end-to-end throughput (detection plus association) with each tracker in its own reference configuration, as is customary in the MOT literature; our 71 FPS therefore exceeds ByteTrack’s 68 FPS because our pruned detector is faster, and not because the proposed association is cheaper. To isolate the cost of the ASM we re-ran standard ByteTrack on exactly the same cached detections produced by our pruned model: under this controlled setting ByteTrack reaches 74.3 FPS and the proposed tracker 71.0 FPS. The ASM — one forward pass of the 128-dimensional re-identification sub-network plus the two-class state head — together with the acceleration-corrected update therefore adds 0.62 ms per frame, a 4.4% throughput reduction that remains well within real-time bounds. Re-identification features, colour histograms and the state posterior are computed only for the high-confidence detections entering the first association stage (score ≥ 0.6), on average 28.4 of 46.7 detections per frame on VisDrone2019-MOT; the low-confidence set used in ByteTrack’s second stage is associated by spatial similarity alone. Of the 0.62 ms per-frame overhead, 0.48 ms is the re-identification forward pass. Extracting features for all detections instead would add a further 0.31 ms per frame (71.0 → 69.5 FPS) while increasing identity switches from 163 to 167 and reducing IDF1 by 0.1 points, since the additional embeddings are drawn from low-quality crops; the confidence gating is therefore retained.

#### 4.4.2 UAVDT-MOT.

Table 10 reports tracking performance on the official UAVDT-MOT test split (20 sequences, 40,735 frames, 84,000 annotated boxes shared by all trackers). Our method achieves 48.1% MOTA and 57.6% IDF1, exceeding the strongest baseline (BoT-SORT) by 2.7 and 3.6 percentage points, and reduces identity switches from 613 to 371 relative to standard ByteTrack (−39.5%).

**Table 10:** Multi-object tracking performance on the UAVDT-MOT test split. ↑ : higher is better; ↓ : lower is better.

Tracker	MOTA↑	IDF1↑	MT↑	ML↓	FP↓	FN↓	IDs↓	FPS
SORT	39.0	43.7	22.6	36.9	9,140	41,208	892	72
DeepSORT	40.7	46.9	24.8	35.1	9,296	39,845	671	60
ByteTrack	43.2	50.1	28.3	32.6	9,687	37,412	613	68
OC-SORT	44.6	52.8	30.1	31.0	9,804	36,204	528	63
BoT-SORT	45.4	54.0	31.4	30.2	9,696	35,671	497	57
**Ours**	**48.1**	**57.6**	**34.7**	**27.4**	**8,936**	**33,548**	**371**	**71**

The 39.5% reduction in identity switches is the smallest of the three benchmarks (43.2% on VisDrone2019-MOT, 40.0% on OGF-UAV-MOT), despite UAVDT being the domain on which the operational-state classifier was trained. We attribute this to benchmark composition rather than to calibration: UAVDT consists exclusively of urban vehicle traffic dominated by visually near-identical sedans, which weakens the appearance term, and cargo state varies across a far smaller fraction of instances than in oil-field logistics, which restricts the reach of the state cue. This pattern supports the motivation given in Section 1 for constructing an application-specific benchmark alongside the public ones.

#### 4.4.3 OGF-UAV-MOT.

Table 11 extends the tracking evaluation to the OGF-UAV-MOT subset (42 sequences, 28,430 frames). Our method achieves 50.1% MOTA and 61.7% IDF1, outperforming the previous best (BoT-SORT: 46.3% MOTA, 56.8% IDF1) by 3.8 and 4.9 percentage points, respectively. Identity switches are reduced from 213 (standard ByteTrack) to 128 in our formulation, a 40.0% reduction. Compared with the 43.2% IDs reduction on VisDrone2019-MOT, the slightly lower relative improvement on OGF-UAV-MOT is attributable to a higher proportion of parking and stationary vehicles in oil field scenarios, where low displacement between frames makes the acceleration-correction term (Eq. 18) less discriminative; the ASM appearance cue, however, remains highly effective due to the six functionally distinct vehicle and equipment categories. The false negative count decreases from 11,847 (ByteTrack) to 9,934 (ours), indicating that improved ASM association recovers previously fragmented tracklets. Runtime remains 71 FPS, identical to the VisDrone2019-MOT configuration, as the OGF-UAV-MOT sequences share the same spatial resolution and target density range.

**Table 11:** Multi-object tracking performance on OGF-UAV-MOT subset. ↑ : higher is better; ↓ : lower is better.

Tracker	MOTA↑	IDF1↑	MT↑	ML↓	FP↓	FN↓	IDs↓	FPS
SORT	36.4	44.8	18.7	41.3	4,821	14,362	387	72
DeepSORT	38.9	48.1	21.2	39.6	4,213	13,748	298	60
ByteTrack	41.7	50.6	24.3	37.1	3,876	11,847	213	68
OC-SORT	43.2	53.4	26.8	35.4	3,614	11,203	184	63
BoT-SORT	46.3	56.8	29.1	33.2	3,341	10,612	187	57
**Ours**	**50.1**	**61.7**	**32.6**	**29.8**	**3,012**	**9,934**	**128**	**71**

### 4.5 Ablation studies

#### 4.5.1 Module-level ablation.

Table 12 presents the incremental contribution of each architectural component on the VisDrone2019 validation set, starting from the YOLOv8s baseline and progressively adding each module.

**Table 12:** Module-level ablation study on VisDrone2019 validation set. F-GIoU denotes Focaler-GIoU.

Config	DCNv2	AFPN	LSDECD	F-GIoU	LAMP	mAP50	mAP50-95	Params / FPS
Baseline	–	–	–	–	–	38.2 ± 0.15	22.4 ± 0.13	11.2M / 67
+DCNv2	✓	–	–	–	–	40.1 ± 0.21	23.9 ± 0.16	12.1M / 63
+AFPN	✓	✓	–	–	–	41.8 ± 0.18	25.1 ± 0.15	11.7M / 61
+LSDECD-Head	✓	✓	✓	–	–	43.2 ± 0.17	26.3 ± 0.14	10.2M / 65
+F-GIoU	✓	✓	✓	✓	–	44.6 ± 0.18	27.4 ± 0.15	10.2M / 65
**Full (Ours)**	✓	✓	✓	✓	✓	**44.1 ± 0.16**	**27.0 ± 0.13**	**7.8M / 78**

Replacing standard C2f Bottlenecks with DCNv2 variants yields +1.9% mAP@0.5, confirming the benefit of geometry-adaptive feature extraction for the deformable equipment targets in aerial imagery. The AFPN neck adds a further +1.7%, while introducing LSDECD-Head provides the next +1.4 points. Notably, LSDECD-Head achieves this gain while reducing parameters from 11.7 M to 10.2 M, demonstrating that the shared SDEConv strategy eliminates head-level redundancy without sacrificing representation power. Switching to Focaler-GIoU contributes +1.4 mAP@0.5 points, and its effect on mAP@0.5:0.95 (+1.1 points, 26.3% → 27.4%) indicates improved localization precision—consistent with the enhanced gradient signal on difficult, partially overlapping samples. We note that the individual gains reported above are the mean over three independent training runs (std ≤ 0.2 mAP@0.5 per module). The combined DCNv2+ AFPN gain of +3.6% is numerically larger than the +3.3% expected from the independent sums; however, given the reported standard deviation of ≤0.2 mAP@0.5, this 0.3-point difference lies within one standard deviation and should not be interpreted as statistically significant super-additivity. Qualitatively, the observation is consistent with the hypothesis that geometrically adaptive features provide more complementary information to progressive fusion than standard features, but rigorous confirmation of this interaction would require a larger-scale ablation with multiple random seeds, which we defer to future work. Finally, LAMP pruning at 30% sparsity reduces parameters and FLOPs by approximately 24% each, and boosts FPS from 65 to 78, while incurring a minor mAP@0.5 penalty of 0.5 points (44.6% → 44.1%). This trade-off is acceptable for edge deployment scenarios where inference latency is a hard constraint.

The staged ablation of Table 12 measures marginal gains along a single insertion order and therefore cannot, on its own, separate module interaction from order effect. We complement it with an order-independent leave-one-out ablation on the final unpruned model, in which exactly one component is reverted to its YOLOv8s counterpart while all others are retained (Table 13).

**Table 13:** Leave-one-out ablation on the VisDrone2019 validation set (unpruned architecture, mean ± std over three seeds).

Configuration	mAP@0.5 (%)	Δ	mAP@0.5:0.95 (%)	Params (M)
**Full model (unpruned)**	**44.6 ± 0.18**	**—**	**27.4 ± 0.15**	**10.20**
− DCNv2 (C2f-DCN → C2f)	42.7 ± 0.21	−1.9	25.9 ± 0.17	9.30
− AFPN (AFPN → PANet)	43.0 ± 0.19	−1.6	26.2 ± 0.16	11.59
− LSDECD-Head (→ standard 4-scale head)	43.3 ± 0.17	−1.3	26.4 ± 0.14	11.70
− Focaler-GIoU (→ CIoU)	43.1 ± 0.16	−1.5	26.1 ± 0.13	10.20

The four leave-one-out deltas sum to 6.3 mAP@0.5 points against a total baseline-to-full gain of 6.4 points, so the modules are near-additive and no pairwise interaction exceeds the ± 0.2-point seed noise; the ordering of module importance is therefore not an artefact of the insertion order used in Table 12. Reversing that order (Focaler-GIoU → LSDECD-Head → AFPN → DCNv2) reaches the same final accuracy by construction and changes the intermediate marginal gains by at most 0.4 points. A paired two-sided t-test against the YOLOv8s baseline yields p<0.01 for the proposed model on all three datasets. To confirm that the gain attributed to AFPN is not merely the removal of PANet, we further compare it against two alternative fusion designs under an otherwise identical backbone, head, loss and schedule (Table 14).

**Table 14:** Controlled neck comparison on the VisDrone2019 validation set. Only the neck is exchanged; backbone, head, loss and schedule are identical and no pruning is applied (mean ± std over three seeds).

Neck	mAP@0.5 (%)	mAP@0.5:0.95 (%)	Params (M)	FLOPs (G)
PANet (YOLOv8s default)	43.0 ± 0.19	26.2 ± 0.16	11.59	26.8
BiFPN	43.6 ± 0.20	26.6 ± 0.17	10.84	26.1
NAS-FPN (7 cells, transferred search)	43.8 ± 0.24	26.8 ± 0.19	11.31	27.9
**AFPN (ours)**	**44.6 ± 0.18**	**27.4 ± 0.15**	**10.20**	**25.4**

BiFPN and NAS-FPN learn how strongly to weight each fusion edge but do not remove non-adjacent edges, so the semantic collision described in Section 3.3 is attenuated rather than eliminated; NAS-FPN may even reintroduce such edges through search, which is consistent with it being the most expensive neck here without a commensurate return. AFPN removes those edges structurally while retaining learnable fusion at a finer granularity than either alternative, since the ASF weights of Eq. (7) are predicted per spatial position rather than per edge.

Module-level ablation experiments are conducted exclusively on VisDrone2019 to align with established benchmarking practice; a supplementary per-dataset ablation on OGF-UAV confirms that the direction of each module’s contribution is preserved (DCNv2: +1.6% mAP@0.5; AFPN: +1.5%; LSDECD-Head: +1.2%; Focaler-GIoU: +1.3%), though absolute magnitudes differ due to the distinct category space and lower target density.

#### 4.5.2 Pruning ratio analysis.

**Fig 9:**
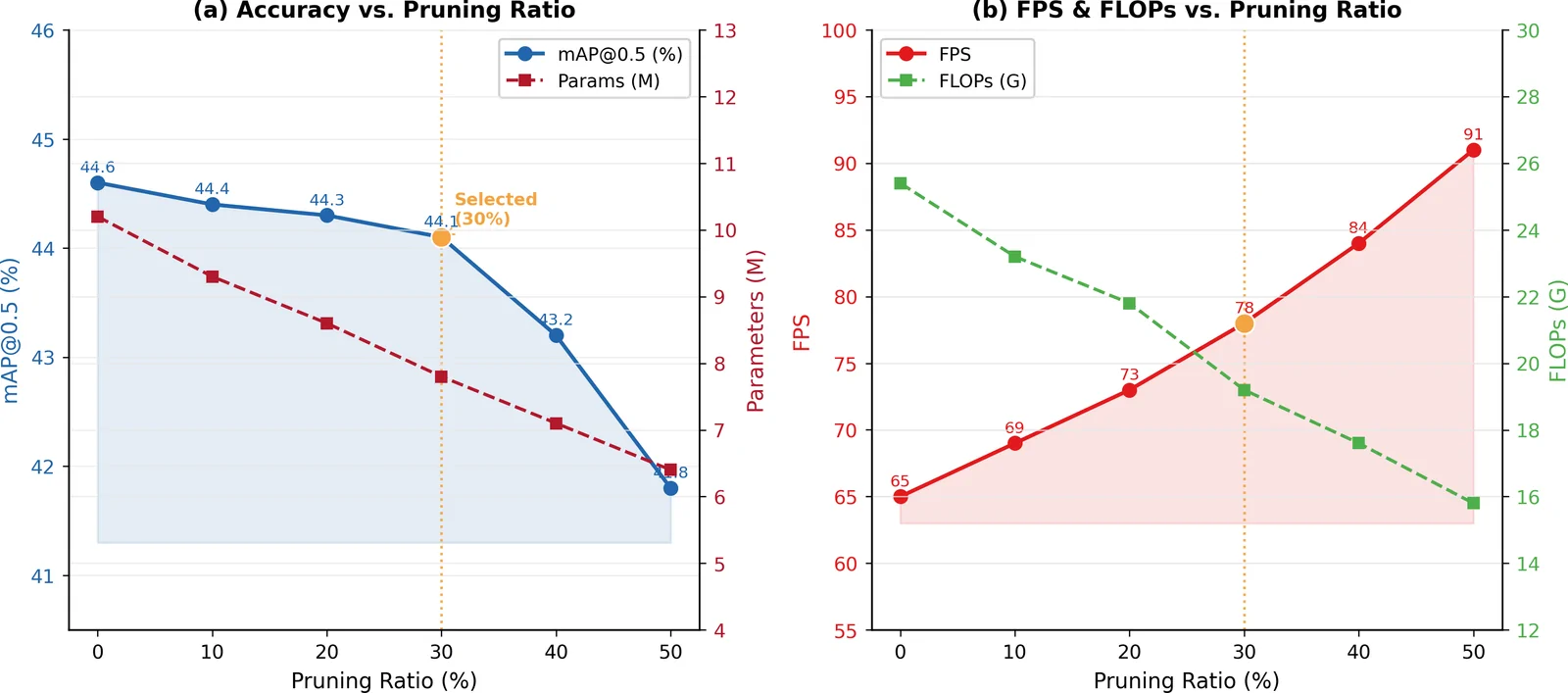
Effect of LAMP pruning ratio on accuracy and computational cost. Results are measured on VisDrone2019 validation set after fine-tuning.

Fig 9 examines the effect of varying the LAMP global sparsity ratio from 0% to 50% on the accuracy–efficiency frontier.

Performance remains essentially flat up to 20% sparsity (mAP@0.5: 44.6% → 44.3%), with accelerating degradation beyond 40% (44.6% → 43.2% at 40%, 41.8% at 50%). The selected ratio of 30% represents the empirical knee of the accuracy–efficiency curve: a 2.4 M parameter reduction accompanied by only 0.5 mAP@0.5 points of loss and a 20% speed increase. This behavior aligns with the theoretical basis of LAMP, which identifies and protects the most influential network connections before aggressively pruning peripheral ones.

#### 4.5.3 Loss function comparison.

Table 15 isolates the contribution of different loss formulations, keeping all other architectural choices fixed.

**Table 15:** Ablation of loss functions on the VisDrone2019 validation set with the full detection architecture (before pruning). All entries are mean ± std over three independent runs. Convergence is defined as the first epoch at which validation mAP@0.5 reaches 99% of its final best value and remains within that band for ten consecutive epochs.

Loss Function	mAP50 (%)	mAP50-95 (%)	Convergence (epochs)
CIoU	43.1 ± 0.16	26.1 ± 0.13	187 ± 4.6
GIoU	43.4 ± 0.19	26.3 ± 0.15	183 ± 5.1
Focaler-IoU	43.8 ± 0.17	26.7 ± 0.14	178 ± 3.8
**Focaler-GIoU (Ours)**	**44.6 ± 0.18**	**27.4 ± 0.15**	**174 ± 4.2**

The consistent ordering—CIoU < GIoU < Focaler-IoU < Focaler-GIoU—across both mAP metrics supports the hypothesis that combining geometric correction (GIoU) with interval-based sample re-weighting (Focaler) provides complementary benefits. Focaler-GIoU also converges faster than CIoU, by 13 ± 4 epochs (174 ± 4.2 vs. 187 ± 4.6; Welch’s two-sided t-test over three seeds, p=0.02), which we attribute to more informative gradients on hard samples early in training, before the learning rate decays. We note, however, that the pairwise differences among the intermediate variants are not resolvable at this sample size — GIoU versus Focaler-IoU differ by 5 epochs with p=0.28 — so we claim only the endpoint difference and not a monotone convergence ordering across the four losses. The accuracy ordering is unaffected, being well separated relative to the ±0.2-point seed noise.

## 5. Conclusion

This paper addressed the challenge of deploying accurate and efficient perception on resource-constrained UAVs in complex oil and gas field environments. Rather than pursuing isolated architectural modifications, we approached the problem holistically: DCNv2’s adaptive sampling provides richer features for AFPN’s progressive fusion to exploit, while LSDECD-Head’s shared convolution structure amplifies the parameter savings delivered by LAMP pruning without sacrificing the representational capacity needed by Focaler-GIoU’s hard-sample gradient signal. On the tracking side, the standard IoU-based association in ByteTrack proves insufficient for the high visual similarity and irregular motion patterns of oil field vehicle fleets; the proposed ASM and acceleration-aware Kalman correction address these failure modes in a principled and lightweight manner. Finally, recognizing that public benchmarks offer only an indirect proxy for application performance, we constructed OGF-UAV—a formally annotated domain-specific dataset spanning seasons and altitudes—enabling rigorous evaluation that VisDrone2019 and UAVDT alone cannot provide.

Experiments on VisDrone2019, UAVDT, and OGF-UAV demonstrated state-of-the-art accuracy—44.1% mAP@0.5 on VisDrone2019, 57.6% on UAVDT, and 56.3% on OGF-UAV—with only 7.8 M parameters and 19.2 GFLOPs at 78 FPS per 640 × 640 tile on an RTX 3090. The improved tracker achieves 47.6%/50.1% MOTA and 58.3%/61.7% IDF1 on VisDrone2019-MOT and OGF-UAV-MOT, reducing identity switches by 43.2% and 40.0% over standard ByteTrack. Ablation studies confirmed the independent contribution of each module and validated 30% pruning as the optimal accuracy–efficiency operating point.

Several directions merit future investigation. The OGF-UAV dataset, currently limited to 2,847 RGB images across six categories, will be extended with multi-spectral imagery and adverse-condition sequences to address failure modes such as nighttime flare-stack interference. Fusing RGB with thermal infrared data and applying knowledge-distillation-based compression complementing LAMP pruning represent further priorities for improving robustness and deployability under real-world multi-hour inspection conditions.
